# A review of fossil scorpion higher systematics

**DOI:** 10.7717/peerj.18557

**Published:** 2024-12-06

**Authors:** Jason A. Dunlop, Russell J. Garwood

**Affiliations:** 1 Museum für Naturkunde, Leibniz Institute for Evolution and Biodiversity Science, Berlin, Germany; 2 Department of Earth and Environmental Sciences, University of Manchester, Manchester, United Kingdom; 3 Natural History Museum, London, United Kingdom

**Keywords:** Scorpiones, Fossils, Higher classification, Families, Arachnids, Arachnopulmonata

## Abstract

Scorpions (Arachnida: Scorpiones) are a diverse and widespread arachnid order with a rich and deep fossil record. Here we review the, sometimes complex, historical development of fossil scorpion higher classification. We present a chronological account of family and genus names, together with an overview of higher taxa as potential clade names. In 1884 Thorell & Lindström divided scorpions based on whether the legs were short and pointed (Apoxypoda) or ended in paired claws (Dionychopoda). Pocock in 1911 used the morphology of the ventral mesosomal sclerites, which could either be bilobed (Lobosterni) or of a modern configuration (Orthosterni). Petrunkevitch in 1949 attached importance to a putative first opisthosomal tergite being present (Protoscorpionina) or absent (Euscorpionina). Kjellesvig-Waering in 1986 recognised four major groups (Holosternina, Meristosternina, Lobosternina and Bilobosternina) based on the shape of the ventral mesosomal sclerites. The Stockwell/Jeram schemes from the 1980s and 1990s proposed a cladistic progression from early branching lineages, for which the names Protoscorpiones and Palaeoscorpiones were used, towards Scorpiones *sensu stricto* defined by the presence of book lungs and coxapophyses. Scorpiones was further divided into Mesoscorpionina and Neoscorpionina. Neoscorpions were characterised by a reduced number of lateral eye lenses and comprise the paleosterns, with marginal lung spiracles, and orthosterns with spiracles in the middle of the sternite. We briefly discuss the merits of these alternatives and present a summary of the current higher classification of scorpions. Forty-three extinct family groups are currently recognised, and of the 24 living families seven have fossil representatives. Including *incertae sedis* taxa, there are 76 extinct genera and five extant genera with fossil representatives. Both modern parvorders, Buthida and Iurida, were potentially present in the Triassic. Buthidae, Chaerilidae, Chactidae and perhaps Hormuridae have been reported from the Cretaceous. Euscorpiidae are known from the Palaeogene and Scorpionidae has potential (but unconfirmed) records from the Neogene. Given the complexity of this history and the present taxonomy of the group, we hope this contribution provides a first step towards simplifying fossil scorpion systematics.

## Introduction

**Figure 1:**
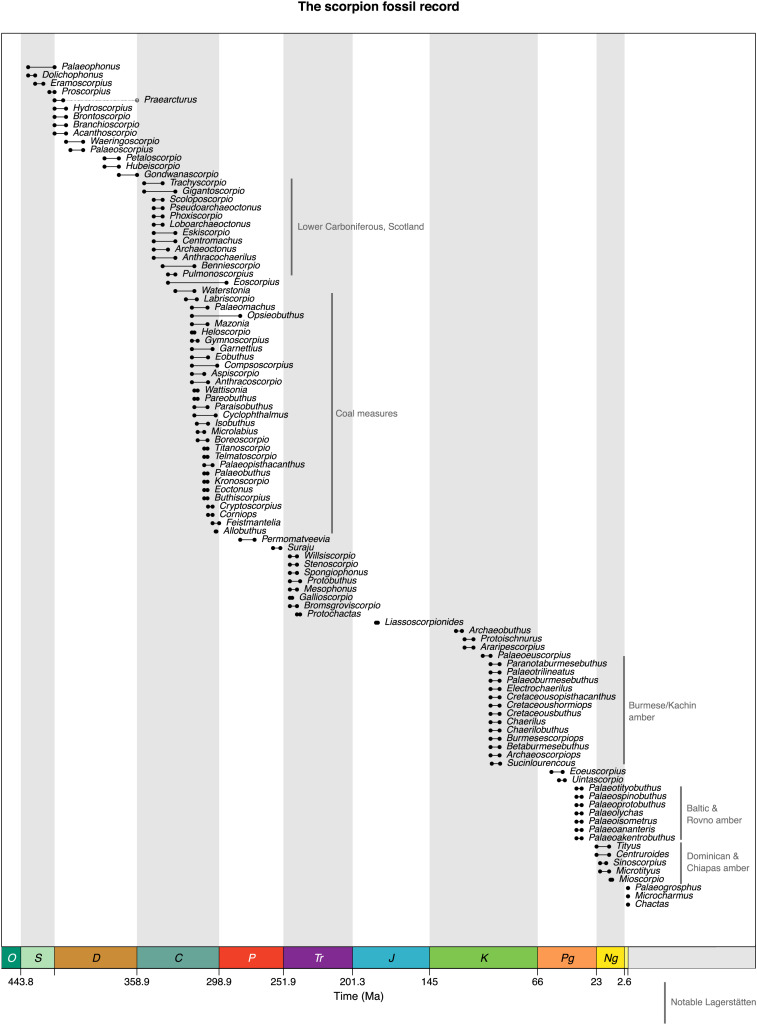
A range through time graph showing the fossil record of the Scorpiones based on records from the palaeobiology database. The concentration of Palaeozoic representatives is of note, as it is unusual for the arachnids.

**Figure 2:**
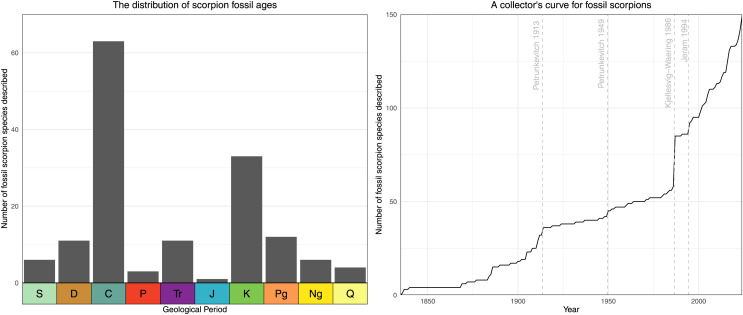
The scorpion fossil record through time. A histogram showing the distribution of accepted fossil scorpion species through geological time (left), and a collector’s curve showing the description of accepted species through time with notable contributions marked on (right).

**Figure 3:**
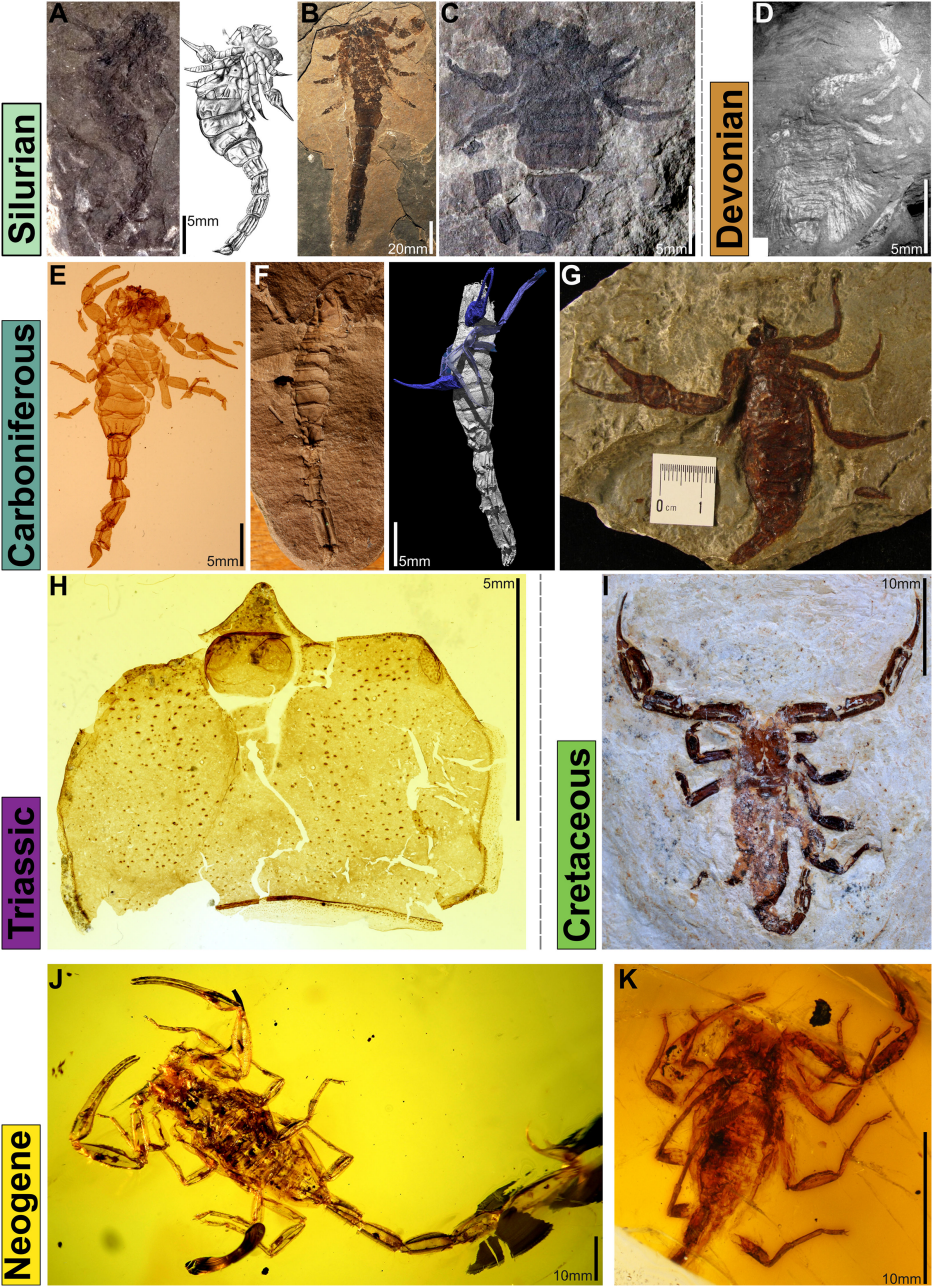
Notable fossil scorpion species. (A) *Palaeophonus caledonicus* Hunter, 1886 (Dick Institute, Kilmarnock, UK) from the mid-Silurian (Llandovery to Wenlock) of Lesmahagow, Scotland, UK, image courtesy of Lyndsay C. Jess, and by permission of East Ayrshire Council/East Ayrshire Leisure Trust, and a reconstruction from Pocock (1901). (B) mid-Silurian *Eramoscorpius brucensis* Waddington, Rudkin & Dunlop, 2015 from Canada. (C) *Proscorpius osborni* Whitfield (1885b), Yale Peabody Museum (YPM IP 545850); photo by Jessica Utrup 2019. (D) Lower Devonian *Waeringoscorpio hefteri* Størmer (1970, image source Poschmann et al., 2008). (E) *Pulmonoscorpius kirktonensis* from the Lower Carboniferous of the UK (courtesy of Andrew Jeram). (F) *Compsoscorpius buthiformis* from the Upper Carboniferous of the UK (left: courtesy of Lorenzo Prendini, AMNH; right: Legg et al., 2012). (G) Carboniferous taxon *Cyclophthalmus senior* from the Yale Peabody Museum collections (YPM IP 029827), photo by Jessica Utrup 2013. (H) *Mesophonus perornatus* from from the Triassic of the UK (courtesy of Lorenzo Prendini, AMNH). (I) *Protoischnurus axelrodorum* from the Cretaceous Crato Formation, Brazil (courtesy of Christian Neumann, Berlin). (J) *Centruroides knodeli* from Neogene Dominican amber (courtesy of Wilson Lourenço, Paris). (K) *Tityus azari* from Neogene Dominican Amber (courtesy of Wilson Lourenço, Paris).

Scorpions (Arachnida: Scorpiones) are an ancient lineage, dating back at least 435 million years to the mid-Silurian (Laurie, 1899; Fig. 1). Compared to other arachnid orders, the scorpion fossil record is surprisingly rich throughout most of their geological history (Figs. 2, 3). Currently, there are 155 accepted species of fossil scorpion in the literature (see Appendix), nine of which belong to incertae sedis taxa), and until quite recently there were more species of Palaeozoic (538.8–251.9 million years ago (Ma)) scorpions than there were younger Mesozoic (251.9–66.0 Ma) and Cenozoic (66.0–0 Ma) fossils combined (Legg et al., 2012; Fig. S1). Recent discoveries, particularly in amber (reviewed in Lourenço, 2015c, 2016a, 2023), have increased our knowledge of Mesozoic and Cenozoic scorpions, but almost half of the currently accepted fossil scorpion species are still Palaeozoic in age. This time period is of particular interest for several reasons. It documents the earliest scorpion lineages, with some of the Silurian (443.8–419.2 Ma) and Devonian (419.2–358.9 Ma) forms differing notably from living species in their leg morphology (Thorell & Lindström, 1885) or in bearing putative external gills (Størmer, 1970). The Palaeozoic also includes the transition to anatomically modern-looking forms which appear by the Late Carboniferous (ca. 315 Ma; Vogel & Durden, 1966). There is also a recurring question about whether the first scorpions were aquatic or terrestrial animals (Peach, 1885; Dahl, 1921): for a critical review see Howard et al. (2019). This, in turn, has implications for our understanding of arachnid phylogeny (Giribet, 2018; Sharma, Ballesteros & Santibáñez-López, 2021) and the number of times that arachnids made the transition from water onto land (see Discussion in Garwood & Dunlop, 2023). In this light, the scorpions are an important group of terrestrial arthropods for understanding critical evolutionary transitions more broadly, and the assembly of the arthropod fauna in modern terrestrial ecosystems.

The higher systematics of extant scorpions has attracted considerable controversy, with Prendini & Wheeler (2005) arguing that some studies of their relationships lack rigour; see Fet & Soleglad (2005) for a response. Living scorpions are now usually divided into two major lineages (or parvorders): Buthida and Iurida (*e.g.*, Soleglad & Fet, 2003, Sharma et al., 2015; Santibáñez-López et al., 2023). While there is molecular data suggesting that modern families may have begun to diverge from one another as early as the Carboniferous (*e.g.*, Loria et al., 2022, fig. 3) the oldest putative examples of the two parvorders come from the Triassic (ca. 247.2–242 Ma), albeit in extinct families (Lourenço & Gall, 2004; Magnani, Stockar & Lourenço, 2022; Viaretti, Bindellini & Dal Sasso, 2023). The oldest scorpions referred to living family groups are Cretaceous. A species from the ca. 115 Ma Crato Formation of Brazil was referred to Chactidae (Menon, 2007). A second Crato species either belongs in the extant family Hormuridae or an extinct relative Protoischnuridae; see Carvalho et al. (2023) for a recent account and discussion of its affinities. Fossils from the slightly younger Burmese amber (ca. 99 Ma) have been assigned to Chaerilidae (Santiago-Blay et al., 2004) and perhaps even the modern genus *Chaerilus* Simon, 1877 (Xuan, Cai & Huang, 2023a). Others were assigned, at least tentatively, to Buthidae (Lourenço & Velten, 2022). Cretaceous ambers also host several fossils placed in extinct families belonging either to the superfamily Buthoidea, or to the wider parvorder Buthida; but see Baptista et al. (2006) for a critique of some of these placements. Iurida is also present in Cretaceous ambers, with fossils assigned to the disputed Protoischnuridae (see above) and an extinct family resembling the living Euscorpiidae (Lourenço, 2003).

All Cenozoic fossil scorpions described so far can be placed in living families. There are several extinct genera of Buthidae in Eocene (ca. 37.7–33.9 Ma) Baltic amber (*e.g.*, Lourenço & Weitschat, 2000) and the probably contemporary Ukrainian Rovno amber (Lourenço & Weitschat, 2009), some of which were reassigned to the recently revalidated family Ananteridae (Ythier, 2024). A handful of modern scorpion genera have been recorded in the Miocene Dominican Republic (ca. 20–15 Ma) and Mexican Chiapas (ca. 24 Ma) ambers; for a recent account of the age of Chiapas amber see Riquelme et al. (2024). These ambers have yielded the Neotropical buthid genera *Centruroides* Marx, 1890, *Microtityus* Kjellesvig-Waering, 1966, *Tityus* C.L. Koch, 1836 and *Rhopalurus* Thorell, 1876 (*e.g.*, Schawaller, 1979; Lourenço, 2009, 2014; Riquelme et al., 2015). Some of these placements merit confirmation; for example the putative *Rhopalurus* in amber is well outside its modern distribution range. There are also examples of scorpions in Quaternary (<2.58 Ma) copal–a subfossil resin which is essentially a precursor to amber–from Madagascar (Lourenço, 1996) and Colombia (Lourenço & Weitschat, 2005a). These again include specimens assignable to living and fossil genera in modern families.

One of the challenges for understanding scorpion evolution is the absence of a modern scheme of higher systematics for the fossils, particularly the Palaeozoic taxa. Authors still have to draw primarily on the comprehensive, but problematic, posthumous monograph of Kjellesvig-Waering (1986), in which many new families and superfamilies were proposed under the presumption that most Palaeozoic and Mesozoic species were habitually aquatic. Kjellesvig-Waering’s scheme is thus rather ‘top heavy’ (see below) with many (super) families represented by a single species and little implied resolution of relationships between higher taxa. Although some progress towards a more modern, cladistic, classification of the fossil scorpions has been made (Jeram, 1994a, 1994b, 1998), these results were not always translated into formal changes in their higher systematics, and most recent publications have employed informal groupings. For this reason Kjellesvig-Waering’s scheme was adopted in the Catalog of the scorpions of the world (1758–1998) (Fet et al., 2000) and has been largely followed, albeit reluctantly, in the updated fossil arachnid species list of Dunlop, Penney & Jekel (2023). It is also worth mentioning that there have been few attempts to integrate fossil and living higher taxa into a unified classification, notable exceptions being the *Treatise on Invertebrate Paleontology* (Petrunkevitch, 1955) and the thesis of Stockwell (1989).

Here, we aim to review the historical development of the higher classification of fossil scorpions as an aid, and hopefully a prelude, to future phylogenetic studies. In particular, we wish to document the origins of family names, as well as the names (and original diagnostic characters) of available higher taxa which could be used for clades recovered in future analyses.

## Materials and Methods

Fossils and their nomenclature were reviewed from the literature, including the *Treatise on Invertebrate Paleontology* (Petrunkevitch, 1955), the extensive monograph of Kjellesvig-Waering (1986), the catalogue of Fet et al. (2000) and published summaries by Dupre (2017, 2020, 2024). Two putative fossil scorpion genera have been shown to be misidentifications. *Tiphoscorpio* Kjellesvig-Waering, 1986 was based on fragments of a Devonian arthropleurid millipede (Shear & Selden, 1995). *Parioscorpio* Wendruff et al., 2020 is Silurian in age, and was even proposed as the oldest scorpion, but appears to be either an indeterminate arthropod or perhaps belongs to an extinct group called the cheloniellids (Anderson et al., 2021; Braddy & Dunlop, 2021). Neither genus is considered further here. For living taxa, we primarily draw on the higher classification in Santibáñez-López et al. (2023: table 1).

In order to create range plots, and as the basis for derived images, we downloaded age range data for all scorpion species from the Paleobiology Database (Uhen et al., 2023) in May 2024. Data were imported into the R programming language (R Core Team, 2024), and the data was cleaned, correcting - for example - typographic errors derived from the literature and removing data points resulting from outstanding taxonomic issues (*Eoscorpius* has been used historically as a genus name for both fossil scorpions and sablefish). We used the palaeoverse package (Jones et al., 2023) to create Fig. 1 from these data, and modified the tax_range_time function of that package to allow grouping by family (all relevant data and the associated R script are available in the electronic supplement). All other taxonomy figures were then constructed from the outputs of the modified tax_range_time function using Inkscape, adding suprafamilial ranks as required. For this we primarily employed a discretised Viridis colour scheme (Garnier et al., 2024).

## Early work

The first written record of a fossil scorpion comes from the German naturalist August Friedrich Schweigger, who figured a specimen in resin in an addendum to a monograph on corals (Schweigger, 1819). The fossil was subsequently named by his compatriot Friedrich Holl, who placed it in the living genus *Scorpio* and thus implicitly the family Scorpionidae. Based on Holl’s (1829) description and figure it probably belongs in a different modern family, Buthidae, but the material is apparently lost and it is not entirely clear whether Schweigger’s material was from Eocene Baltic amber or a younger copal deposit; see Lourenço & Weitschat (1996) and Dunlop & Jekel (2008) for further discussion.

Carboniferous Coal Measures scorpions were first mentioned by Kašpar Maria Count Sternberg in a published lecture (Sternberg, 1835). Often regarded as the father of palaeobotany, Sternberg’s scorpion comes from Cholme, a village in the Březina district of Bohemia where the family owned property including coal mines. The Bohemian scorpions were described by his compatriot and collaborator August Josef Corda, who introduced the genus names *Cyclophthalmus* Corda, 1835 and *Microlabis* Corda, 1839. The latter was actually described as an “Afterskorpion”, which is an older German word for a pseudoscorpion: a different arachnid order. The next fossils to be described were from the Coal Measures of Mazon Creek in Illinois, USA, published by the American geologists Fielding Bradford Meek and Amos Henry Worthen, who introduced the genera *Eoscorpius* Meek & Worthen, 1868a and *Mazonia* Meek & Worthen, 1868b.

A second amber scorpion, this time demonstrably from Palaeogene Baltic amber, was described by the German naturalist and arachnologist Anton Menge. It was assigned by Menge (1869) to *Tityus*, a modern Neotropical genus of the family Buthidae. This makes little biogeographical sense and although Menge’s fossil is believed lost, Lourenço & Weitschat (1996) made a convincing case for its probable misidentification at genus level. The English geologist Henry Woodward described a large fossil from Early Devonian of England as a crustacean under the genus name *Praearcturus* Woodward, 1871. It was later reinterpreted as an almost metre long scorpion, however the material is incomplete and lacks unequivocal scorpion features such that doubts remain about its arachnid affinities (Simon Braddy, 2024, personal communication). Woodward (1876) described an unequivocal scorpion from the Carboniferous Coal Measures of England in Meek & Worthen’s genus *Eoscorpius*. The same genus was also used by another British geologist, Benjamin Neeve Peach, for some Carboniferous scorpions from Scotland (Peach, 1883).

The first family-group name for fossil scorpions was raised by the American palaeoentomologist Samuel H. Scudder, who proposed Eoscorpionidae (Scudder, 1884)–sometimes rendered as Eoscorpiidae–to accommodate *Cyclophthalmus* (here treated as a senior synonym of *Microlabis*), *Eoscorpius* and *Mazonia*. The original diagnosis of Eoscorpionidae referred to the presence of a pair of sternal plates between the coxae of the second pair of legs, which Scudder (1884) regarded as the principal difference between living and Carboniferous species, alongside some possible differences in pectine morphology. Another Bohemian Coal Measures genus, *Anthracoscorpio* Kušta, 1885, was described by the Czech palaeontologist Johann (Jan) Kušta.

## The first silurian scorpions

**Figure 4:**
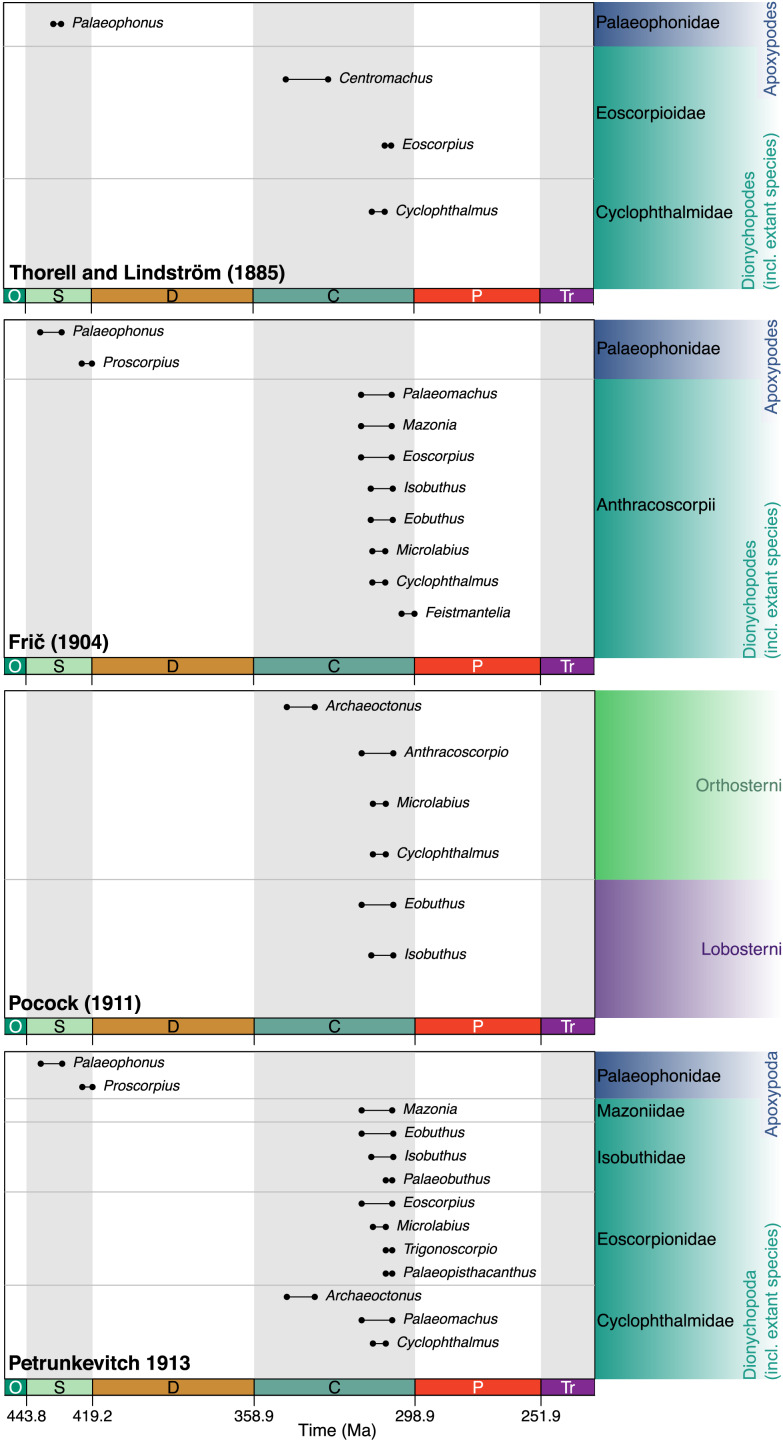
Early taxonomic schemes incorporating fossil scorpions. Ranges for each fossil genus are shown based on data derived from the Palaeobiology Database, but feature only the taxa that had been described when any given scheme scheme was published. The scheme of Thorell & Lindström (1885) divides scorpions into two suborders based on the presence (Dionychopodes) or absence (Apoxypodes) of paired claws, and Antonín Frič (Fritsch, 1904) followed this. The scheme of Reginald Innes Pocock (1911) primarily incorporates taxa from the British Coal Measures, and divides the scorpions into Lobosterni (with bilobed mesosomal sternites) and Orthosterni (with a more modern-looking sternal configuration). Finally, that of Alexander Petrunkevitch (1913) follows the subordinal scheme of Thorell & Lindström and Frič.

Around this time the Swedish arachnologist Tamerlan Thorell and palaeontologist Gustaf Lindström described the first Silurian scorpion. It comes from Gotland in Sweden and was placed in a genus *Palaeophonus* Thorell & Lindström, 1884a–initially misspelt *Palaeophoneus*, see also a mention under the correct spelling in Thorell & Lindström (1884b)–prior to its formal description by Thorell & Lindström (1885). In this article the authors also proposed a basic division of scorpions into two suborders (Fig. 4). Apoxypodes was defined on a tapering tarsus, without claws, and was restricted to their new Silurian family Palaeophonidae created for *Palaeophonus*. The suborder Dionychopodes was defined on a sub-cylindrical or clavate tarsus bearing a pair of movable claws. This second suborder was further subdivided into two series. Anthracoscorpii was defined on a carapace which is usually pointed at the front and median eyes close to the anterior margin of the carapace. It encompassed the known Carboniferous species, which Thorell & Lindström (1885) split into two families. Scudder’s Eoscorpioidae (note emended spelling) was accepted for *Eoscorpius* and a new genus, *Centromachus*, created for the British Carboniferous species described by Woodward and Peach. Eoscorpioids were defined as scorpions with median eyes situated between the lateral eyes. A new family, Cyclophthalmoidae, was introduced for *Cyclophthalmus* and defined on very large eyes positioned in front of the lateral eyes. The second series, Neoscorpii, was defined on a carapace with a truncate or emarginate anterior margin and median eyes in a more posterior position. It encompassed all living scorpions, plus Menge’s (now lost) amber fossil.

Another Silurian scorpion, this time from New York State in the USA, was described by the American palaeontologist Robert Paar Whitfield. First assigned to Thorell & Lindström’s *Palaeophonus* by Whitfield (1885a), it was subsequently raised to its own genus *Proscorpius* Whitfield, 1885b. In the same year Scudder (1885) contributed the arachnid chapter to the German textbook *Handbuch der Palaeontologie*, adopting a modified version of Thorell & Lindström’s scheme. Scudder recognised two suborders: Anthracoscorpii for the Palaeozoic species and Neoscorpii for the living and amber species. Anthracoscorpii were defined as scorpions with the front of the carapace produced and eyes in an anterior position. Two families were accepted, Palaeophonidae (for *Palaeophonus*) defined on features like the pointed tarsi and Eoscorpionidae with paired tarsal claws. Three eoscorpionid subfamilies were recognised. The new taxon Proscorpionini (for *Proscorpius*) was defined on its large median eye tubercle with small eyes at the front of the carapace. Eoscorpionini (for *Eoscorpius*, its putative synonym *Mazonia* and for *Centromachus*) was defined on smaller eyes. Finally, Cyclopthalmini (for *Cyclophthalmus* and its putative synonym *Microlabius*) was defined on a very large median eye tubercle and correspondingly large median eyes.

Scudder (1885) also cited a report by Peach (1885) of a new Silurian *Palaeophonus* from Scotland. It was formally described and named a year later by the Scottish geologist John Robert Hunter(-Selkirk) (Hunter, 1886). It should be mentioned that the Canadian geologist George Frederic Matthew also assigned a Carboniferous fossil from New Brunswick in Canada to *Palaeophonus*. The fossil in Matthew’s (1895) widely overlooked article is probably not a scorpion and was treated as a *nomen dubium* by Dunlop & Garwood (2023).

## Frič’s monograph

The Czech naturalist Antonín Frič–who usually published as Anton Fritsch–wrote several studies of fossil arachnids with a focus on the Coal Measures of Bohemia, culminating in his extensive monograph *Palaeozoische Arachniden* (Frič 1904). He adopted Thorell & Lindström’s basic division into the suborders Apoxypodes (without paired claws) and Dionychopodes (with paired claws; Fig. 4). Apoxypodes comprised here the family Palaeophonidae with the Silurian genera *Palaeophonus* and *Proscorpius*. Dionychopodes comprised a single family Anthracoscorpii (note the non-standard ending) including the known Carboniferous genera *Cyclophthalmus*, *Microlabius*, *Eoscorpius*, *Mazonia* and *Centromachus*. Three new anthracoscorpiid genera from Bohemia were added: *Eobuthus* Frič, 1904, *Feistmantelia* Frič, 1904 and *Isobuthus* Frič, 1904.

## The first triassic scorpions

The British geologist Leonard Johnston Wills was the first to describe scorpion fossils from the Mesozoic, namely from the Triassic ‘Keuper’ of the Bromsgrove district in the West Midlands of England. Largely based on fragments of cuticle macerated from the matrix, he proposed the genus *Mesophonus* Wills, 1910 in the family Mesophonidae and an order [*sic*] Mesophonidea. An explicit diagnosis of the new family was not given, but Wills (1910) contrasted at some length the morphology of these fossils and living scorpions. Most significantly, differences include the presence in fossil taxa of median eyes on a projection at the front of the carapace, multi-facetted lateral eyes, and putative book-lung spiracles opening at the posterior margins of the ventral opisthosomal sclerites as opposed to lying in the middle of these sclerites as in the orthostern condition seen more derived fossils as well as the living taxa.

## Pocock’s lobosterns and orthosterns

A significant figure in fossil arachnid research was the British zoologist Reginald Innes Pocock, who wrote a monograph focussed on the British Carboniferous Coal Measures fauna. As well as reviewing previous classification schemes, Pocock (1911) introduced the names Lobosterni and Orthosterni for scorpions (Fig. 4). Lobosterns were defined as having bilobed sternal plates on the ventral opisthosoma. Orthosterns were defined as having a modern configuration of these same sternites; the usage would be later revised to restrict this to taxa with visible lung spiracles within the sternite. In the same monograph he raised the genus *Palaeomachus* Pocock, 1911 for a previously described species from the Coal Measures of Mansfield in England and *Archaeoctonus* Pocock, 1911 for two previously described species from near Edinburgh in Scotland. In Pocock’s (1911) scheme *Eobuthus* and *Isobuthus* were lobosterns, *Cyclophthalmus*, *Archaeoctonus*, *Anthracoscorpio* and perhaps *Microlabius* were orthosterns. Other genera known at that time were considered of uncertain affinities and family groups were not recognised in Pocock’s monograph.

## Petrunkevitch’s first monograph

Another significant fossil arachnid worker was the Russian-born, later USA-based, Alexander Petrunkevitch. His first foray in the subject was a monograph (Petrunkevitch, 1913) focussed on the North American Coal Measures fauna. Like Frič, he adopted Thorell & Lindström’s suborders with the names modified here to Apoxypoda and Dionychopoda respectively. In Petrunkevitch’s scheme (Fig. 4) Apoxypoda still comprised a single Silurian family, Palaeophonidae, for *Palaeophonus* and *Proscorpius*. Dionychopoda included four Carboniferous families. Isobuthidae was proposed as a new family by Petrunkevitch (1913) and defined as scorpions in which coxae of the fourth pair of legs abut the genital opercula. It encompassed *Isobuthus, Eobuthus* and a new genus *Palaeobuthus* Petrunkevitch, 1913. Cyclophthalmidae (the name was proposed by Thorell and Lindström, not Petrunkevitch) was defined as scorpions with a normal coxal arrangement, median eyes not close to the anterior edge of the prosoma and a broad chela with short fingers. It encompassed *Cyclophthalmus, Palaeomachus, Archeoctonus* and the newly proposed *Eoctonus* Petrunkevitch, 1913 from Mazon Creek. Eoscorpionidae was again defined on a normal coxal arrangement and median eyes not close to the anterior edge of the prosoma, but here with a slender chela with long fingers. It encompassed *Eoscorpius, Microlabius* and the newly proposed *Trigonoscorpio* Petrunkevitch, 1913 and *Palaeopisthacanthus* Petrunkevitch, 1913, both again from Mazon Creek. Finally, a new family Mazoniidae was defined by Petrunkevitch (1913) as having median eyes close to the anterior edge of the prosoma and contained only *Mazonia*.

## The first unequivocal devonian scorpion

The German palaeontologist Walter Maximilian Lehmann described the first fossil scorpion from the Devonian, namely from the Hunsrück Slate of Germany. Lehmann had previously worked at a factory producing X-ray equipment (Gerth, 1961) and later applied these techniques to the Hunsrück fossils, including the scorpion. The Hunsrück example is of particular note for the debate about terrestrialisation in that it comes from an environment widely interpreted as a marine deposit as evidenced by the co-occurrence of groups like echinoderms, trilobites and sea spiders. Lehmann (1944) commented on the fact that the fossil’s excellent preservation implied that if it were a terrestrial animal it must have lived close to the coast with only a short transport distance to the burial place. He proposed a new genus *Palaeoscorpio* Lehmann, 1944 and while the family name Palaeoscorpionidae has been assigned to Lehmann (1944) too, this is in fact a widely perpetuated error. It appears that Petrunkevitch (1953) first used the family group name, see also comments in Kutscher (1971), which he diagnosed on a longitudinal swelling along the body.

## Wills’ revisits the triassic scorpions

Wills (1947) published a second study of the British Triassic scorpions, adding detailed descriptions of several new and previously known species. The new genus *Spongiophonus* Wills, 1947 was proposed, but not explicitly referred to a family. Wills (1947) further elucidated the differences between his Triassic scorpions and living species, including the presence of multi-facetted lateral eyes, an anterior projection of the carapace bearing the median eyes, the marginal position of the book-lung spiracles on the sternites and some details of the body ornamentation. He further suggested some similarities between these scorpions and modern buthids such as the shape of the sternum and spines on the margins of the lung spiracles.

## Petrunkevitch’s second monograph

In his second major fossil arachnid monograph Petrunkevitch (1949) re-examined the British Coal Measures fauna and also proposed several fundamental changes to the higher systematics of scorpions (and other arachnids). Petrunkevitch now recognised two scorpion suborders: Protoscorpiones, in which first tergite of the opisthosoma was interpreted as present (albeit sometimes concealed) to yield a preabdomen of eight segments, and Euscorpiones in which the first opisthosomal tergite was completely lost to give the modern-looking preabdomen with only seven visible tergites. Under the protoscorpions Petrunkevitch (1949) recognised the Silurian family Palaeophonidae, which he defined on short legs with pointed tarsi and the fourth coxae abutting the anterior sides of a pentagonal sternum. Palaeophonids included *Palaeophonus* (which he now treated as a senior synonym of *Proscorpius*) and a new genus, *Dolichophonus* Petrunkevitch, 1949, for a Scottish Silurian species described previously by Laurie (1899). The second protoscorpion family was the Carboniferous Mazoniidae, restricted to the genus *Mazonia*, which was diagnosed on having a fully exposed first opisthosomal tergite and somewhat longer legs.

Under the euscorpions Petrunkevitch (1949) recognised four families, all from the Carboniferous. Isobuthidae was defined on the third leg coxae abutting the sternum and the fourth leg coxae abutting the genital operculum and included *Eobuthus* and *Isobuthus*. A new family, Archaeoctonidae, was defined by Petrunkevitch (1949) on fairly short legs and pedipalp claws. It included the genera *Archaeoctonus*, *Palaeomachus* and *Eoctonus*. Cyclophthalmidae was defined on longer legs and pedipalps and a bluntly pointed carapace and included *Centromachus* and *Cyclophthalmus*. Eoscorpionidae was defined again on longer legs and pedipalps, but here with a more rounded or emarginated carapace. This became the most diverse fossil scorpion family including *Anthracoscorpio*, *Trigonoscorpio*, *Eoscorpius* and *Palaeopisthocanthus*, as well as seven new genera from the British Coal Measures: *Alloscorpius* Petrunkevitch, 1949, *Compsoscorpius* Petrunkevitch, 1949, *Europhthalmus* Petrunkevitch, 1949, *Lichnophthalmus* Petrunkevitch, 1949, *Lichnoscorpius* Petrunkevitch, 1949, *Typhlopisthacanthus* Petrunkevitch, 1949 and *Typhloscorpius* Petrunkevitch, 1949. He excluded the Czech genera *Feistmantellia* and *Microlabius* from this scheme on the grounds of a lack of characters.

## The first jurassic scorpion

The first, and still the only, record of a Jurassic scorpion was published by the German geologist Arnold Bode from the Lias of Hondelage near Braunschweig in Germany. *Liassoscorpionides* Bode, 1951 was not originally assigned to a family, although it would eventually be mooted as the youngest scorpion belonging to an aquatic lineage (see below). Restudy of the original material by Dunlop, Kamenz & Scholtz (2007) suggested much over-interpretation of what is actually a rather poorly-preserved specimen, best treated as Scorpiones *incertae sedis*, an opinion also shared by Dubinin (1962); see also Appendix.

## Petrunkevich’s third monograph

A subsequent study by Petrunkevitch, now including the re-examination of fossil arachnids from other European countries, largely followed his 1949 scheme and continued to use the suborders Protoscorpiones and Euscorpiones. In the protoscorpions the all-important first opisthosomal tergite was now described as being “concealed under the carapace and faintly visible in outline” (Petrunkevitch, 1953), which does not inspire confidence in its veracity. The family Palaeophonidae was retained for *Palaeophonus*, a new family Dolichophonidae was established by Petrunkevitch (1953) for *Dolichophonus* and the now revalidated *Proscorpius* based on their more modern looking legs compared to the pointed legs of palaeophonids. Mazoniidae was retained for *Mazonia*.

Among the euscorpions, Lehmann’s Palaeoscorpionidae (for *Palaeoscorpius*) was now included in the suborder, and the family defined on a “median longitudinal bulge along the opisthosoma”, which sounds suspiciously like a preservational character. Archaeoctonidae was retained for *Archaeoctonus* and *Eoctonus* and redefined as having three pairs of coxae abutting the sternum. A new family, Centromachidae, was proposed by Petrunkevitch (1953) for *Centromachus* where coxae III and IV abut the genital opercula. Isobuthidae was retained for *Isobuthus*, *Palaeobuthus* and now also *Microlabius* and again diagnosed on coxae III abutting the sternum and coxae IV abutting the genital opercula. Cyclophthalmidae, with *Cyclophthalmus*, defined on coxae III meeting in front of the sternum and coxae IV abutting the sternum. Eoscorpionidae was again noted as being the most species-rich fossil family and the group closest to modern scorpions in its morphology being diagnosed on an essentially modern coxo-sternal region with coxae III and IV abutting the sternum. The genera *Alloscorpius*, *Compsoscorpius*, *Eoscorpius*, *Europthalmus*, *Palaeopisthacanthus*, *Trigonoscorpio*, *Typhloscorpius* and *Typhlopisthacanthus* were all included here. Two new Carboniferous euscorpionid genera were also added: *Buthiscorpius* Petrunkevitch, 1953 for one of Pocock’s (1911) British species and *Garnettius* Petrunkevitch, 1953 for a species described by Elias (1936) from Kansas in the USA. Finally, Will’s Triassic family Mesophonidae (with *Mesophonus* and *Spongiophonus*) was formally placed among the euscorpions and defined on only the first pair of leg coxae having “maxillary lobes” (*i.e.*, coxapophyses). *Palaeomachus* and *Liassoscorpionides* were listed as Scorpiones *incertae sedis*.

## The *treatise*

**Figure 5:**
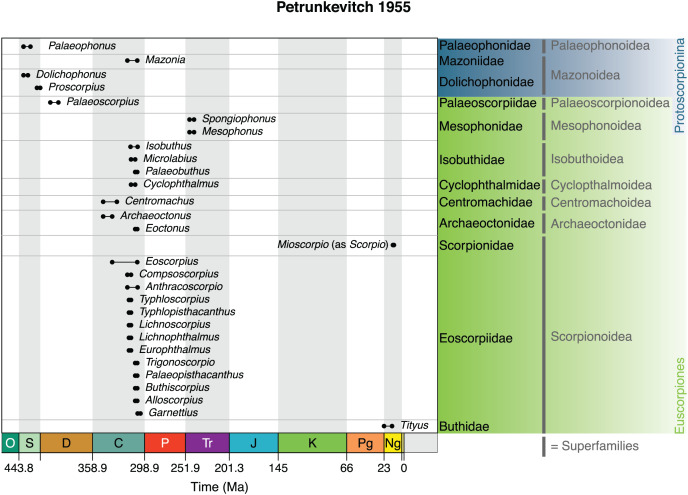
The 1955 scheme of Petrunkevitch. This retained the overall structure of the author’s previous schemes, by splitting the scorpions into the suborders Protoscorpiones and the Euscorpiones, based on the interpretation of opisthosomal segmentation, and also introduced a number of superfamilies.

In the *Treatise on Invertebrate Paleontology* Petrunkevitch (1955) again retained the main divisions from his 1949 monograph with some minor changes to the spelling: *i.e.*, Protoscorpionina, retaining the first opisthosomal tergite, and Euscorpionina in which the first tergite has been lost (Fig. 5). Protoscorpions were divided into two superfamilies. Palaeophonoidea was characterised by thick legs ending in a single pointed claw and included only Palaeophonidae and *Palaeophonus*. By contrast, Mazonoidea was characterised by thinner legs, presumed to have borne a pair of claws, and included Dolichophidae (for *Dolichophonus* and *Proscorpius*) in which the all-important extra first tergite was supposed to be concealed under the carapace and Mazoniidae (for *Mazonia*) in which the extra tergite was described as fully visible.

The euscorpions encompassed the remaining scorpions, here also now divided into superfamilies. Since a couple of living genera were now known to have fossil representatives, at least two modern families were also included in this scheme. Palaeoscorpionoidea was defined again on the “median longitudinal fold” and included only the Devonian family Palaeoscorpiidae and *Palaeoscorpius*. Archaeoctonoidea was defined on the first pair of coxae meeting in front of the sternum and the others abutting the sternum. It was restricted to Archaeoctonidae with *Archaeoctonus* and *Eoctonus*. Scorpionoidea was defined on both the first and second pair of leg coxae having coxapophyses (maxilliary lobes). It included the fossil family Eoscorpiidae, here re-defined on having the coxae of legs III and IV being similar in size to those of legs I and II. This family included most of the Carboniferous genera, as per the 1949 and 1953 monographs. The living families Scorpionidae and Buthidae were also placed in Scorpionoidea and their more elongate leg coxae III and IV were noted, with the two living families differentiated here on the shape of the sternum and the number of lateral eyes.

The euscorpion superfamily Cyclopthalmoidea was defined as having three pairs of coxae meeting in front of the sternum and the fourth pair abutting the sternum. It was restricted to Cyclophthalmidae and *Cyclopthalmus*. Isobuthoidea was characterised by two pairs of coxae in front of the sternum, the third abutting the sternum and the fourth abutting the genital operculum. It contained the family Isobuthidae with *Isobuthus*, *Microlabius* and *Palaeobuthus*. Centromachoidea was characterised by the first pair of coxae in front of the sternum, the second and third abutting the sternum and the fourth abutting the genital operculum. It was restricted to Centromachidae and *Centromachus*. Finally, the Triassic Mesophonoidea was defined as having two pairs of coxae in front of the sternum, only the first having coxapophyses, and book lung spiracles at the margins of the sternites. This superfamily contained Mesophonidae with *Mesophonus* and *Spongiophonus* (listed erroneously as *Spongiotarsus*). For completeness, *Palaeomachus* and *Liassoscorpionides* were again listed as Scorpiones *incertae sedis*.

## Wills’ studies of carboniferous scorpions

In addition to his work on the Triassic scorpions, Wills also wrote two detailed articles on Carboniferous material. Here, he adopted Pocock’s higher classification–as opposed to Petrunkevitch’s–with an initial study on Lobosterni (Wills, 1959) followed by one on Orthosterni (Wills, 1960). In the first article he introduced the genus *Pareobuthus* Wills, 1959 for a Coal Measures scorpion from the Welsh Borderlands. *Lichnophthlmus* material was also included under the lobosterns. In the second article, he described several orthostern scorpions etched from British and North American Coal Measures nodules. These orthosterns included *Buthiscorpius*, and the new genera *Mazoioscorpio* Wills, 1960 from Mazon Creek and *Wattisonia* Wills, 1960 from the English West Midlands. As a supplement to the lobosterns, he also added the genus *Benniescorpio* Wills, 1960 for Peach’s (1883) Scottish scorpion; this now being the fifth genus to which Peach’s fossil had been assigned! None of Will’s genera were formally assigned to a family. Wills (1960) also speculated that the lobostern scorpions were aquatic and had gills, and lived alongside the orthosterns which were fully terrestrial, with the new genus *Mazoioscorpio* possibly transitional between the two major groups. He also commented on the difficulties in establishing a scheme of scorpion higher systematics given both the rarity of fossils and differences in the way in which they are preserved.

## Other post-*treatise* developments

The Russian parasitologist, Vsewolod Borisovich Dubinin, also authored several palaeontological works including a (posthumous) summary of the fossil chelicerates in the volume *Osnovy Paleontologii* (Fundamentals of Palaeontology) (Dubinin, 1962). Here, he favoured Thorell and Lindström’s basic Apoxypodes/Dionychopodes division and also introduced two new family names. Trigonoscorpionidae was raised for *Trigonoscorpio*, and some other Carboniferous genera, but was rejected by Kjellesvig-Waering (1886) as a family of dubious standing. Several of its taxa are now synonyms of *Compsoscorpius*, which is itself of uncertain familial position (Legg et al., 2012). Garnettiidae was raised for Petrunkevitch’s genus *Garnettius*. It remains a valid family and was originally diagnosed on a suite of characters including the short and stout pedipalps. Also of note in a phylogenetic context is his earlier (Dubinin, 1957) proposal, also adopted in the 1962 work, to divide Chelicerata into five classes with the traditional arachnids split across several groups. His Scorpionomorpha, encompassed scorpions, the extinct sea scorpions (Eurypterida), as well as pseudoscorpions, whip scorpions, whip spiders, palpigrades and ricinuleids.

The Norwegian geologist and palaeontologist Leif Størmer made several important contributions to the study of fossil arthropods. In addition to articles on horseshoe crabs and eurypterids, Størmer (1963) briefly reviewed previous fossil scorpion classifications while promoting, like Dubinin, the hypothesis that scorpions were closely related to eurypterids, and the idea that all Silurian and some Carboniferous scorpions were probably aquatic. In the same article he offered a detailed description of *Gigantoscorpio* Størmer, 1963 from the Early Carboniferous of Scotland; an unusually large fossil scorpion suggested as having been up to 35 cm long, with later workers proposing even larger estimates. It was not referred to a family. A key figure in the story of fossil scorpion systematics was Erik Kjellesvig-Waering, who was born in Cuba, but had Norwegian and later American nationality. Better known for his extensive studies of the extinct sea scorpions (Eurypterida), his first article on fossil scorpions was a revision of the Silurian Bertie Waterlime fossils of New York in which he introduced the new genus *Archaeophonus* Kjellesvig-Waering, 1966.

## Gills and giants

Given the debate about whether early scorpions were aquatic or terrestrial, a remarkable discovery from the Early Devonian of Alken an der Mosel in Germany was the first scorpion with direct evidence of gills. *Waeringoscorpio* Størmer, 1970 preserves pairs of filamentous projections from the postero-lateral margins of the mesosoma. It was placed by Størmer in a new family, Waeringoscorpionidae, diagnosed on the fact that all leg coxae surround a pear-shaped sternum. Størmer (1970) further discussed evolutionary trends in the scorpion sternum and interpreted the filamentous regions as true gills, and explicit evidence for an aquatic habitat, albeit perhaps displaced from their supposed original position under a series of eurypterid or horseshoe-crab-like gill opercula. Later studies confirmed the presence of these structures in other fossils assignable to this genus (Brauckmann, 1987; Poschmann et al., 2008). The latter authors interpreted the filamentous elements as structures which genuinely project out from the pleural region of the mesosoma and drew comparisons with the tracheal gills of some modern insects, raising the intriguing possibility that these are respiratory organs in a secondarily aquatic scorpion. It also worth noting the study of Di, Edgecombe & Sharma (2018) who described a rare homeotic modern scorpion–*i.e.*, one where mutation has transformed one structure into a different one–with sternal protrusions associated with a book lung, which the authors suggested could be homologous to a *Waeringoscorpio* gill. Kutscher (1971) briefly redescribed the *Palaeoscorpius devonicus* from the Devonian of Germany, but felt unable to say for sure if it was an aquatic (*i.e.*, gilled) or terrestrial scorpion.

In his second contribution to fossil scorpions, Kjellesvig-Waering (1972) described the free finger of what he interpreted as the pedipalp of a very large scorpion from the Devonian of Worcestershire in England. *Brontoscorpio* Kjellesvig-Waering, 1972 was estimated as having been up to 90 cm in length. It was not assigned to a family group and, if it is a scorpion, it is currently regarded as Scorpiones *incertae sedis* (see Appendix). Since scorpions are not the only arthropods with chelate appendages, this record based on such incomplete material should be treated with caution.

## New genus records

During the 1980s three articles were published describing three new fossil scorpion genera from the three eras of the Phanerozoic. The American palaeobotanist Richard Leary described a new scorpion found among Coal Measures plant remains from Illinois. *Labriscorpio* Leary, 1980 is an interesting late Palaeozoic record in that it appears to retain a plesiomorphic coxo-sternal region, more typical for Silurian or Devonian species, with the coxae abutting quite a large sternum, a lack of coxapophyses on coxae I and II and (as the name implies) an apparent labium in front of the sternum. Leary (1980) did not assign it to a family.

Cenozoic shale-preserved scorpions are rare, and the Chinese palaeoentomologist Yong-Chong Hong described a scorpion from the Shanwang Formation in Shandong Province, China. *Sinoscorpius* Hong, 1983 was referred to the modern family Scorpionidae (Hong, 1983, 1985), but as noted in the catalogue of Fet et al. (2000) there has been no independent confirmation of this assignment.

From the Mesozoic, the Crato Formation of north-eastern Brazil is an important locality preserving a mid-Cretaceous (ca. 115 Ma) fauna which includes numerous insects and arachnids. The Brazilian palaeontologist Déa Regina Bouret Campos described the first Crato scorpion. *Araripescorpius* Campos, 1986 is quite modern-looking, with large, robust pedipalps. Campos (1986) did not assign the original specimen to a family, but the revision of Menon (2007) introduced new material from which she referred the genus to the modern family Chactidae where it remains the oldest member of the group and indeed the oldest scorpion genus assigned to a living family.

## Kjellesvig-waering’s monograph

It is difficult to summarise all the changes proposed in Kjellesvig-Waering’s (1986) monograph, given that it represents such a radical rearrangement, yet it is necessary to attempt this in order to understand the current state of scorpion higher systematics.

**Figure 6:**
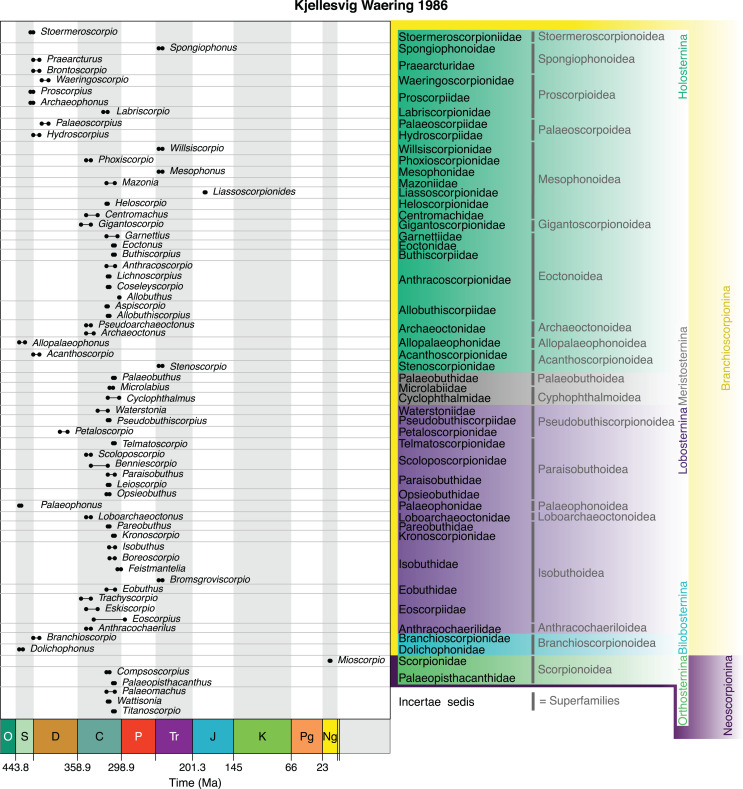
The scheme of Kjellesig-Waering (1986), a significant rearrangement of previous schemes, and the basis of much of today’s fossil scorpion taxonomy. This posthumous publication split the scorpions into Branchioscorpionina and Neoscorpionina based on inferred respiratory physiology.

We should also reiterate that his monograph was published posthumously and the compilers often had to interpret Kjellesvig-Waering’s ideas from his notes. In overview, Kjellesvig-Waering (1986, fig. 1) started from the premise that scorpions and eurypterids shared a common ancestor and that almost all Palaeozoic and Mesozoic scorpions were aquatic and had gill opercula (abdominal plates in his terminology) on the ventral surface of the mesosoma. Kjellesvig-Waering (1986) thus recognised an order Scorpionida divided into the newly introduced suborder Branchioscorpionina, defined as primarily aquatic scorpions retaining gills, and an emended version of Thorell & Lindström’s suborder Neoscorpionina, here defined as scorpions possessing lungs (Fig. 6). The putatively aquatic branchioscorpions were further subdivided into four infraorders: Holosternina, Meristosternina Lobosternina and Bilobosternina based on the way, and degree to which, the abdominal plates were divided. The newly proposed infraorder Holosternina was defined as having five pairs of rectangular, undivided abdominal plates: this observation in fossil scorpions is not trivial as modern scorpions only have four pairs of lung-bearing sternites. The holostern superfamily Proscorpiodea was defined as holosterns with all coxae abutting a large sternum, the first meeting in front of it, but without coxapophyses. It included Proscorpiidae (for *Proscorpius* and *Archaeophonus*), defined as having compound lateral eyes anterolaterally on the carapace and two claws on the legs. Waeringoscorpionidae (for *Waeringoscorpio*) was redefined as having a sternum and labium, and all coxae radiating from the sternum. A new questionable proscorpioid family, Labriscorpionidae (for *Labriscorpio*), was proposed defined by a large pentagonal sternum and subcunate coxae.

The holostern superfamily Stoermeroscorpionoidea was defined by the first pair of coxae in front of the sternum beginning to develop coxapophyses, the second and third coxae abutting the sternum, and the fourth pair the genital operculum. It included the new family and genus Stoermeroscorpioniidae and *Stoermeroscorpio* Kjellesvig-Waering, 1986 from the Silurian Bertie Waterlime of the USA. Allopalaeophonoidea was defined as having three pairs of coxae in front of the sternum, no coxapophyses and short, thick legs. It included the new family Allopalaeophonidae and *Allopalaeophonus* Kjellesvig-Waering, 1986 raised for Hunter’s (1886) Scottish Silurian scorpion. Palaeoscorpoidea was defined by having all four pairs of coxae meeting in front of the sternum and encompassed Palaeoscorpiidae (for *Palaeoscorpius*) defined on a pentagonal sternum. A new family of putative palaeoscorpioids, Hydroscorpiidae, defined by a median eye node in the first quarter of the carapace and leg segments as wide as long. It contained the new genus *Hydroscorpius* Kjellesvig-Waering, 1986 from the Early Devonian of Wyoming, USA.

The holostern superfamily Archaeoctonoidea was defined as having the first pair of coxae meeting in front of the sternum without coxapophyses, the other three abutting the sternum, and short legs. It included the family Archaeoctonidae for *Archaeoctonus* and a new genus *Pseudoarchaeoctonus* Kjellesvig-Waering, 1986 from the Early Carboniferous of Scotland. Acanthoscorpionoidea was similar in having the first pair of coxae in front of the sternum, but here with coxapophyses, and the other three coxae abutting the sternum. It included the new family Acanthoscorpionidae, defined on an ovoid sternum and compound lateral eyes for the new genus *Acanthoscorpio* Kjellesvig-Waering, 1986 from the Devonian of Wyoming, USA, and the new family Stenoscorpionidae diagnosed by a carapace divided into two halves without lateral eyes raised for a new genus, *Stenoscorpio* Kjellesvig-Waering, 1986, created for one of Wills’ (1910) Triassic species. Gigantoscorpionoidea was defined by two pairs of coxae meeting in front of the sternum, without coxapophyses, and the other two coxae abutting the sternum. It encompassed Gigantoscorpionidae as a new family raised for *Gigantoscorpio*.

The holostern superfamily Mesophonoidea was defined by two pairs of coxae with coxapophyses in front on the sternum, the third pair abutting the sternum and the fourth pair the genital opercula. It included Mesophonidae (for *Mesophonus*) redefined on a narrow sternum and median eyes placed well forward. Mazoniidae (for *Mazonia*) was redefined on a subequilateral pentagonal sternum and the second pair of coxapophyses reaching about half the length of the first pair. Centromachidae (for *Centromachus*) was redefined on a small subtriangular sternum. A new family Heloscorpionidae, was defined on one of the typical coxo-sternal patterns and an elongate carapace with a deep ‘V’ shaped sulcus. It included a new genus, *Heloscorpio* Kjellesvig-Waering, 1986, raised for a British Carboniferous species originally described by Woodward (1907). Liassoscorpionidae (for *Liassoscorpionides*) was proposed as a new family defined on centrally positioned median eyes and lateral schizochroal eyes; *i.e.*, compound eyes, but the lenses not touching. A new family, Phoxiscorpionidae, was defined on an elongate hexagonal sternum and a very large pectinal plate. It contained a new genus, *Phoxiscorpio* Kjellesvig-Waering, 1986 from the Early Carboniferous of Scotland. The final mesophonoid family was Willsiscorpionidae, a new family with very forward placed median eyes but no lateral eyes, containing the new genus *Willsiscorpio* Kjellesvig-Waering, 1986 raised for one of Wills’ (1910) Triassic species.

The holostern superfamily Eoctonoidea was defined on the first pair of coxae being in front of the sternum and having coxapophyses where the first pair extend further forward than the second. It included a new family Eoctonidae (for *Eoctonus*) defined small schizochroal eyes and median eyes in the centre of the anterior half of the carapace. Another new family, Buthiscorpiidae (for *Buthiscorpius*) was defined on a similar eye morphology and a median sulcus dividing the carapace into two cephalic cheeks. The new family Allobuthiscorpiidae was defined as probably eoctonoids without lateral eyes and included the new genera *Allobuthiscorpius* Kjellesvig-Waering, 1986 created for one of Wills’ (1960) species and *Aspiscorpio* Kjellesvig-Waering, 1986, created for a Carboniferous specimen from Lancashire in the UK. The new family Anthracoscorpionidae was defined by a large pentagonal sternum and long coxapophyses and included *Anthracoscorpio*, *Lichnoscorpius*, and the new British Carboniferous genera *Allobuthus* Kjellesvig-Waering, 1986 and *Coseleyscorpio* Kjellesvig-Waering, 1986. The remaining eoctonoid family was Garnettiidae (for *Garnettius*) redefined on having fossorial flattened legs with large spurs and serrations.

The final holostern superfamily was Spongiophonoidea defined on the first pair of coxae having short coxapophyses, the second pair without partly abutting the sternum, and coxae III and IV abutting the sternum. The new family Spongiophonoidae (for *Spongiophonus*) was defined by a large, triangular sternum and pustulate ornament. The new family Praearcturidae (for *Praearcturus*) was defined by its very large size and lanceolate, medially divided sternum. *Brontoscorpio* was placed here too based on its size, even though it is only known from a free finger.

The newly proposed infraorder Meristosternina (Fig. 6) was defined as having abdominal plates divided by a median suture. One of its superfamily/family/genus groups was later shown to be a misidentified myriapod (see Introduction). The superfamily Cyphophthalmoidea was defined as having two coxae in front of the sternum, the third abutting the sternum and the fourth abuting the genital operculum. It contained Cyclophthalmidae (and *Cyclophthalmus*). Microlabiidae was proposed as a new family for *Microlabius*. In Palaeobuthoidea the first two pairs of coxae were again in front of the sternum but the third and fourth coxae also abutted the sternum. It contained Palaeobuthidae as a new family for *Palaeobuthus*.

The infraorder Lobosternina adopted Pocock’s name and was redefined as scorpions with gently to deeply bilobed abdominal plates, presumed to cover gill chambers. Palaeophonoidea was proposed for scorpions with short, thick legs and contained Palaeophonidae and *Palaeophonus*. Anthracochaeriloidea was proposed for a new family and genus (Anthracochaerilidae: *Anthracochaerilus* Kjellesvig-Waering, 1986) from the Early Carboniferous of Scotland defined by only very slightly bilobed abdominal plates. Isobuthoidea was defined having two coxae in front of the sternum, the third abutting the sternum and the fourth abuting the genital operculum; note that this is essentially the same as for the meristosternine group Cyphophthalmoidea. Isobuthoids included Isobuthidae, defined on narrow coxapophyses and a pentagonal sternum longer than wide. It included *Isobuthus*, *Feistmantelia* and the new genera *Boreoscorpio* Kjellesvig-Waering, 1986 from Carboniferous of Nova Scotia in Canada and *Bromsgroviscorpio* Kjellesvig-Waering, 1986 from the Triassic of the English West Midlands. Eobuthidae (for *Eobuthus* only) was proposed as a new family, defined on a pyriform sternum and the coxapophyses of coxa 1 being spatulate. Eoscorpiidae was defined on a pentagonal sternum, again spatulate first coxapophyses, and the presence of multifaceted eyes. It encompassed *Eoscorpius* and the new genera *Trachyscorpio* Kjellesvig-Waering, 1986 and *Eskiscorpio* Kjellesvig-Waering, 1986, both from the Early Carboniferous of Scotland. The new family Pareobuthidae (for *Pareobuthus*) was defined on a hexagonal sternum and pectines with an undivided rachis and basal lamella. Kronoscorpionidae was defined on a narrow lacrimiform sternum and elongate, spatulate first coxapophyses and again with multifaceted eyes. It included a new genus *Kronoscorpio* Kjellesvig-Waering, 1986 from Late Carboniferous of Mazon Creek.

The lobostern superfamily Paraisobuthoidea was proposed for scorpions where the coxapophyses reach the anterior margin (of the body), with the first pair crowded out from coming into contact with each other on the midline by the second pair. Here, Paraisobuthidae was defined on a pentagonal sternum and schizochroal lateral eyes. This family included the new Coal Measures genera *Paraisobuthus* Kjellesvig-Waering, 1986 and *Leioscorpio* Kjellesvig-Waering, 1986. A new family Telmatoscorpionidae was defined on a small barrel-shaped sternum and large median, but small compound eyes. It was restricted to the new genus *Telmatoscorpio* Kjellesvig-Waering, 1986 from Mazon Creek. Scoloposcorpionidae was defined as paraisobuthoids lacking lateral eyes and contained *Benniescorpio* and a new genus *Scoloposcorpio* Kjellesvig-Waering, 1986 from the Early Carboniferous of Scotland. Opsieobuthidae was defined on a short wide lacriiform sternum and a well developed so-called prepectinal plate. It included the new genus *Opsieobuthus* Kjellesvig-Waering, 1986 for a species described by Moore (1923) from Indiana in the USA.

The lobostern superfamily Loboarchaeoctonoidea was defined as having the first pair of coxae meeting in front of the sternum, but no coxapophyses, and the other three pairs of coxae abutting the sternum. It contained a single new family, Loboarchaeoctonidae, for the newly proposed genus *Loboarchaeoctonus* Kjellesvig-Waering, 1986 from the Early Carboniferous of Scotland. The final lobostern superfamily was Pseudobuthiscorpionoidea defined by the first pair of coxae abutting the second pair which meet on the midline, with coxae I and II in front of the sternum and II and IV abutting the sternum. It contained the new family Pseudobuthiscorpiidae in which the coxapophyses of the second coxae extend as far forwards as those of the first coxae and with a large pentagonal sternum. It was restricted to the new genus *Pseudobuthiscorpius* Kjellesvig-Waering, 1986 from the Later Carboniferous of the English West Midlands. Another new family Petaloscorpionidae was defined by the second pair of coxapophyses only being about half the length of those of the first coxae. It included the new genus *Petaloscorpio* Kjellesvig-Waering, 1986 from the Late Carboniferous of Quebec in Canada. Finally Waterstoniidae was proposed for having a lacrimiform sternum and the second pair of coxapophyses reaching about two-thirds the length of the first pair. It was restricted to the new genus *Waterstonia* Kjellesvig-Waering, 1986 from the Early Carboniferous of Scotland.

The newly proposed infraorder Bilobosternina (Fig. 6) was defined as scorpions with fully bilobed abdominal plates, in other words two separate, rounded plates on the ventral surface. It contained a single superfamily, Branchioscorpionoidea, with a new family Branchioscorpionidae, and genus *Branchioscorpio* Kjellesvig-Waering, 1986 from the Devonian of Wyoming, USA; the family diagnosed as having abdominal plates covering normal (*i.e.*, orthostern) sternites. Petrunkvitch’s family Dolichophonidae (for *Dolichophonus*) was also included under the bilobosterns on account of a putative bilobed abdominal plate; Kjellesvig-Waering (1986) sketched it preserved next to the body.

Finally, the pulmonate suborder Neoscorpionina contained a single infraorder, Pocock’s Orthosterni, called here Orthosternina (Fig. 6). Here, within the modern superfamily Scorpionoidea, Kjellesvig-Waering (1986) recognised a new family Palaeopisthacanthidae for *Palaeopisthacanthus* and *Compsoscorpius*, defined as scorpions with a well developed prepectinal plate and spatulate coxapophyes on the first coxae. Within the modern family Scorpionidae a new genus *Mioscorpio* was recognised for a Miocene scorpion described previously by Hadži (1931) from the German Swabian Alb. The taxa *Wattisonia*, *Palaeomachus* and a new genus *Titanoscorpio* Kjellesvig-Waering, 1986 were regarded as *incertae sedis*.

## Stockwell’s thesis

**Figure 7:**
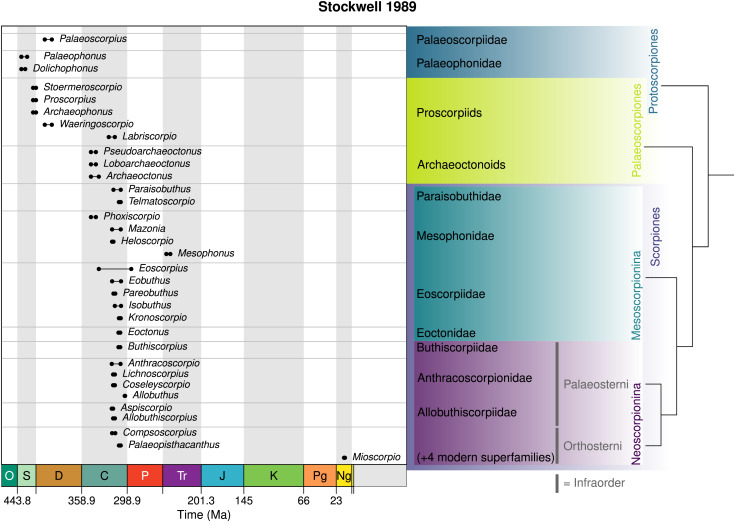
The scheme of Stockwell (1989), based on a cladistic analysis of 137 characters (tree topology shown on the right). This introduced a number of new groups which have sometimes been used informally since.

As noted above, the thesis of the American biologist Scott Stockwell attempted to integrate living and fossil scorpions into a single systematic scheme based on a cladistic analysis of 137 characters (Fig. 7). Kjellesvig-Waering’s groups were not supported, and Stockwell (1989) questioned the usefulness of abdominal plate shape as a character for higher classification. Stockwell proposed raising scorpions to a class, rather than an order, since in his analysis he could not distinguish between eurypterids being the sister-group of scorpions or the non-scorpion arachnids. He also summarised arguments for the earliest scorpions being aquatic.

For the higher classification, Stockwell (1989) recognised a class Scorpionida divided into three orders: Protoscorpiones, Palaeoscorpiones and Scorpiones. His protoscorpions were an emended version of Petrunkevitch’s (1949) name, here characterised by a coxosternal region with coxae I–III surrounding a mouth in front of the sternum and coxae IV abutting the sternum as well as digitigrade legs with short articles. Protoscorpiones encompassed Palaeophonidae, in which Stockwell included *Palaeophonus*, *Allopalaeophonus* and *Dolichophonus* (thus effectively synonymising Allopalaeophonidae and Dolichophonidae) as well as Palaeoscorpiidae for *Palaeoscorpius*.

Palaeoscorpiones was a newly proposed clade name for scorpions with longer legs and better developed tarsal claws. All leg coxae abut the sternum and only the first pair (if any) meet in front of the sternum. Palaeoscorpions were subdivided into two informal groups. Proscorpiids were effectively equivalent to the family Proscorpiidae (considered here a senior synonym of Waeringoscorpionidae, Labriscorpionidae and Stoermeroscorpionidae) with *Proscorpius*, *Archaeophonus*, *Waeringoscorpio*, *Labriscorpio* and *Stoermeroscorpio*. Archaeoctonoids were equivalent to the family Archaeoctonidae (with Loboarchaeoctonidae as its synonym) and the Scottish Carboniferous genera *Archaeoctonus*, *Pseudoarchaeoctonus* and *Loboarchaeoctonus*.

Scorpiones *sensu* Stockwell comprised those taxa with the first and second pair of leg coxae meeting in front of the sternum and forming a preoral chamber (*i.e.*, bearing coxapophyses/maxillary lobes). The leg articles are also laterally compressed and the tarsus lies flat on the substrate in a plantigrade stance. Several genera (see Stockwell, 1989 for the complete list) were considered *incertae sedis* on account of their poor or incomplete preservation and included the usual suspects like *Praearcturus* and *Liassoscorpionides*. The remaining members of Scorpiones were split between two suborders: a new group Mesoscorpionina and an emended version of Thorell & Lindström’s Neoscorpionina. Mesoscorpions were defined as having coxae IV abutting the genital operculum (not the sternum), lateral eyes multifaceted where known and median eyes on an anterior projection of the carapace. It included three families. Mesophonidae (with Mazoniidae Heloscorpionidae and Phoxiscorpionidae as synonyms) incorporated *Mesophonus*, *Mazonia*, *Heloscorpio* and *Phoxiscorpio*. Eoscorpiidae (with Eobuthidae, Isobuthidae, Pareobuthidae and Kronoscorpionidae as synonyms) included *Eoscorpius*, *Eobuthus*, *Isobuthus*, *Pareobuthus* and *Kronoscorpio*. Finally Paraisobuthidae (with Telmatoscorpionidae as its synonym) included *Paraisobuthus* and *Telmatoscorpio*.

Neoscorpionina *sensu* Stockwell was diagnosed on coxae IV abutting the sternum, not the genital operculum, a reduced number of lenses in the lateral eyes (when preserved), and a posterior displacement of the median eyes. It was further divided into a new group, Paleosterni, and Pocock’s Orthosterni. Paleosterns were characterised by book lung spiracles at the margins of the sternites and lateral eyes with eight lenses (where preserved). It included *Eoctonus*, *Buthiscorpius*, *Allobuthiscorpius*, *Aspiscorpio*, *Anthracoscorpio*, *Lichnoscorpius*, *Allobuthus* and *Coseleyscorpio*; with Stockwell suggesting that Kjellesvig-Waering’s (1986) family groups could be retained here. Orthosterns were characterised by book lung spiracles opening within the sternite, rather than at its margins, and lateral eyes with only 2–5 lenses. Three fossil genera were recognised here as Orthosterni *incerate sedis* (*Palaeopisthacanthus*, *Compsoscorpius* and *Mioscorpio*), with the living scorpions divided into four superfamilies: Buthoidea, Chactoidea, Vaejovoidea and Scorpionoidea. Even though Stockwell’s thesis was not formally published, some of his nomenclature was adopted in the later literature (see below).

## The oldest asian scorpion

The German palaeontologist Dieter Walossek and colleagues described a new genus from the Late Devonian of Hubei Province in China. *Hubeiscorpio* Walossek, Li & Brauckmann, 1990 remains the oldest record of an Asian fossil scorpion. Walossek, Li & Brauckmann (1990) felt unable to place it in any higher group based on the characters preserved, but did note the slender leg tarsi as a distinctive feature. In a wider context, the short metatarsus and longer tarsus ending in a pair of claws suggest a condition similar to that of the Silurian genus *Proscorpius* rather than legs with a longer metatarsus as are typically seen in younger fossils and living species. *Hubeiscorpio* is currently placed as Scorpiones *incertae sedis*; see also Appendix.

## Jeram’s initial studies

**Figure 8:**
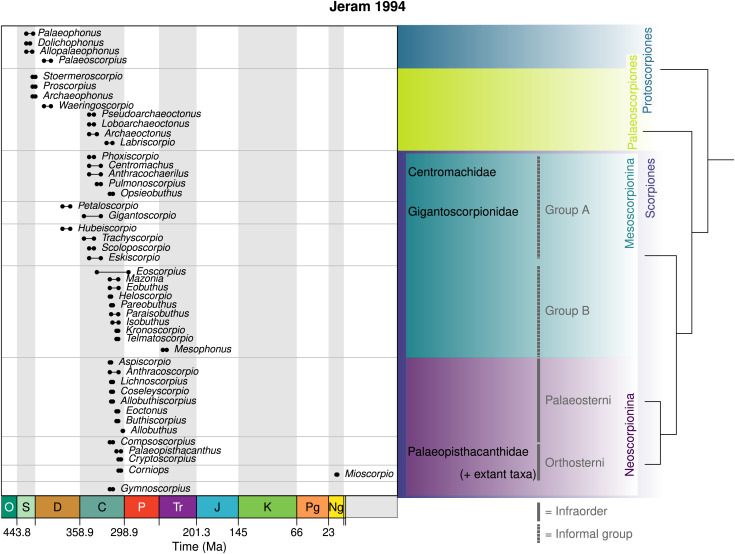
The schemes of Jeram (1994a, 1994b) combined. This was based in part on a cladistic analysis (tree shown right), and incorporates a number of informal groupings. This is largely in keeping with the scheme of Stockwell (1989), and eschews taxonomic considerations.

In 1994 the British palaeontologist Andrew Jeram published two significant articles on fossil scorpions (summarised in Fig. 8). In the first of these, he described *Pulmonoscorpius* Jeram, 1994*a* from the Early Carboniferous of East Kirkton in Scotland. This remarkable species preserves unequivocal evidence for book lungs (see also Jeram, 1990), and includes a near complete specimen with a body length of almost 30 cm, plus fragments suggesting even larger animals. It was assigned to the family Centromachidae. Jeram (1994a) briefly reviewed the Kjellesvig-Waering and Stockwell classification schemes, largely adopting the latter with its basic division of Scorpionida into Protoscorpiones, Palaeoscorpiones and Scorpiones. Major character transformations were highlighted in a summary cladogram (Jeram, 1994a, fig. 1). Scorpiones *sensu* Stockwell was defined here on characters such as book lungs, laterally compressed leg articles and the presence of coxapophyses. It was again divided into Mesoscorpionina, with authorship assigned to Stockwell (but no unique defining synapomorphies) and Neoscorpionina defined by a reduced number of lateral eye lenses, median eyes shifted posteriorly and a long pre-anal segment. The neoscorpions comprised Palaeosterni and Orthosterni, with orthosterns defined by spiracles opening within the sternites and modern patterns of trichobothria, *i.e.*, fine, sensory hairs which occur in taxonomically specific patterns on the scorpion pedipalp. In detail, Jeram (1994a) assigned his new genus plus *Opsiebuthus*, *Anthracochaerilus* and *Phoxiscorpio* to the mesoscorpion family Centromachidae. Under the family Gigantoscorpionidae he recognised *Gigantoscorpio* and *Petaloscorpio*. The genera *Hubeiscorpio*, *Scoloposcorpio*, *Eskiscorpio* and *Trachyscorpio* were listed as Mesoscorpionina *incertae sedis*.

In the second article focusing on fossil orthosterns Jeram (1994b, text-fig. 1) recognised a series of stem group Carboniferous genera accumulating crown group synapomorphies. Orthosterni was again defined on the position of the lung spiracle and trichobothria of the pedipalp. Here Palaeopisthacanthidae was redefined as having four ventral trichobothria on the pedipalp manus and trichobothrium ‘eb’ on the fixed finger. It encompassed *Palaeopisthacanthus*, *Compsoscorpius* and the new genus *Cryptoscorpius* Jeram, 1994b from Lone Star Lake in the USA. Two further genera were described from cuticle fragments from this locality: *Corniops* Jeram, 1994b as Orthosterni *incertae sedis* and *Gymnoscorpius* as Neoscorpionina *incertae sedis*.

## Extinct buthid genera from the palaeogene

The Eocene Green River Formation in Colorado, USA is a shale deposit which has long been known for fossil insects. A scorpion from here was described by Michael Perry and assigned to a new, extinct genus *Uintascorpio* Perry, 1995. In the original study it was compared to living members of the family Vaejovidae. Later revision by Santiago-Blay, Soleglad & Fet (2004), and references therein, suggested that it is actually an extinct genus of Buthidae, but the authors felt unable to determine which of the living buthid genera it is most closely related to.

Putative Baltic amber scorpions include Holl’s (1829) problematic record of a “*Scorpio*” (see above). Another amber species was assigned by the German arachnologist Anton Menge to the modern Neotropical genus *Tityus*. As noted above it is likely that Menge’s (1869) species is misplaced, but as with Holl’s name the original type material appears to be lost. Its status was discussed in the first of several articles on Baltic amber scorpions by the French arachnologist Wilson Lourenço and the German palaeontologist Wolfgang Weitschat. They commented on the fact that Baltic amber scorpions seemed to show Old World, rather than New World, affinities also proposed a new extinct genus of buthid, *Palaeolychas* Lourenço & Weitschat, 1996, for a new scorpion in this amber, which was interpreted as being similar to the widespread modern genus *Lychas*.

## Jeram’s second phylogeny

In a further contribution, expanding his work into scorpion phylogeny, Jeram (1998) offered a cladistic analysis of the Silurian and Devonian taxa based on 26 morphological characters. As in Stockwell’s thesis, Kjellesvig-Waering’s higher taxa were not recovered, with the resulting tree suggesting the following classification scheme (Jeram, 1998: table 2). The Hunsrück genus *Palaeoscorpius* emerged as sister group to all other scorpions as it was thought to retain gnathobasic limbs, similar to those of horseshoe crabs and eurypterids. This was followed by a series of family groups treated here as plesion taxa; which can be taken to mean earlier branches characterised by plesiomorphic character states. Palaeophonidae was characterised by a median emargination of the carapace and included *Palaeophonus*, *Allopalaeophonus* and *Dolichophonus*. Proscorpiidae lacked a clear defining apomorphy (Jeram speculated that in may require further subdivision) and included the Silurian to Carboniferous genera *Proscorpius*, *Archaeophonus*, *Stoermeroscorpio*, *Waeringoscorpio*, *Archaeoctonus*, *Pseudoarchaeoctonus*, *Loboarachaeoctonus*, *Labriscorpio* and *Hydroscorpius*. Praearcturidae was restricted to *Praearcturus* and some undescribed material. The next branch was the plesion genus *Branchioscorpio* followed by the larger Mesoscorpionina clade (see above) including *Acanthoscorpius* and several other genera as per Jeram (1994a). Finally a series of Carboniferous palaeostern scorpions were recognised, leading up to the Carboniferous orthostern genera as per Jeram (1994b) plus the scorpion crown-group. Although this scheme implied that several family groups recognised by Kjellesvig-Waering were synonyms, these changes were only formalised later (see below).

## New genera in copal and amber

Lourenço (1996) described a subfossil scorpion from Madagascan copal in the living genus *Tityobuthus* Pocock, 1893, but later introduced a new extinct genus *Palaeogrosphus* Lourenço, 2000 to accommodate it. Lourenço & Weitschat (2000) continued their studies of Eocene Baltic amber scorpions, recognising three new extinct genera in the family Buthidae. *Palaeotityobuthus* Lourenço & Weitschat, 2000 was interpreted as being similar to *Tityobuthus*. *Palaeoakentrobuthus* Lourenço & Weitschat, 2000 was interpreted as being similar to the modern genus *Akentrobuthus* Lamoral, 1976. Finally, *Palaeoprotobuthus* Lourenço & Weitschat, 2000 was regarded as being possibly intermediate between buthids and microcharmids; although there is debate in the literature about whether Microcharmidae should be considered a valid family (see *e.g.*, Lowe & Kovařík, 2022, and references therein) with the family having been explicitly synonymised with Buthidae by Volschenk, Mattoni & Prendini (2008). Soon after, another new genus *Palaeoananteris* Lourenço & Weitschat, 2001 was introduced, with similarities to the modern genus *Ananteris* Thorell, 1891.

We note that the holotype of Lourenço’s (1996) copal species originated from a private collection, albeit perhaps in the meantime it has been donated to the museum of the Geological-Palaeontological Institute of the University of Hamburg. In fact, quite a lot of studies continue to describe fossil scorpion species from copal and amber based on type specimens in private collections (*e.g.*, Santiago‐Blay & Poinar, 1988; Lourenço, 2001; Lourenço & Beigel, 2015; Lourenço & Velten, 2024). This may raise issues about the accuracy of data associated with such material, and there are also obvious potential problems about how future researchers will be able to trace and access specimens if the hosting collection is broken up and/or sold on after the current owner’s death. For further discussion and critique of this topic see Riquelme et al. (2015).

## Cretaceous scorpions

The ca. 125 Ma (Cretaceous: Barremian) Lebanese amber is one of the oldest inclusion-bearing resins. Lourenço (2001) described the first scorpion from this deposit in a new, extinct genus *Archaeobuthus* Lourenço, 2001. A new family, Archaeobuthidae, was raised to accommodate it and placed within the wider superfamily Buthoidea (see also Appendix). Archaeobuthids were diagnosed as having small and bulky pectines, small and rounded spiracles, a unique pattern of strong, knife-shaped spines on the fingers of the pedipalp claw and a distinctive pattern of trichobothria on the pedipalp (see also below).

The Brazilian palaeontologist Maria da Gloria P de Carvalho teamed up with Wilson Lourenço to describe another, well-preserved scorpion from Crato Formation of Brazil. *Protoischnurus* Carvalho & Lourenço, 2001 was assigned to a new, extinct family Protoischnuridae which was diagnosed on a suite of characters including a pentagonal sternum, moderately large pectines without fulcra and a type ‘C’ trichobothrial pattern on the pedipalp claw. Carvalho & Lourenço (2001) considered protoischnurids to be similar to the modern families Scorpionidae and Hemiscorpiidae (here under the older name Ischnuridae). Menon (2007) later synonymised Protoischnuridae with Hemiscorpiidae based on a central suture on the carapace ending in an inverse ‘Y’ shape, the position of the *est* trichobothrium, an internal well-developed projection of the pedipalp patella, the position of carinae on the pedipalp chelae and a sternum longer than wide. It should be noted that the extinct family was retained as valid by, *e.g*., Lourenço & Velten (2021); see also below.

The ca. 99 Ma (Cretaceous: Cenomanian) Burmese amber of Myanmar has become an increasingly rich source of information about mid-Cretaceous animals living on what may, at that time, have been an island which split off from Australia and later collided with Asia. The first scorpion from Burmese amber was described in a new, extinct genus *Palaeoburmesebuthus* Lourenço, 2002. The holotype specimen is only known from the metasoma (or tail) and was not originally assigned to a family (but see below), being simply listed by Lourenço (2002) as Buthoidea *incertae sedis*.

French amber from Archingeay is of similar (mid-Cretaceous) age to Burmese amber. A scorpion described from here based on an isolated pedipalp was assigned to a new extinct genus *Palaeoeuscorpius* Lourenço, 2003. Despite its incompleteness, a new extinct family within the superfamily Chactoidea was proposed: Palaeoeuscorpiidae. It was diagnosed on a unique pattern of trichobothria on the pedipalp, similar to the type ‘C’ *sensu* Vachon (1974), but with a reduced number of trichobothria especially on the patella. It was interpreted as being closest to members of the living genus *Euscorpius*.

## Integrating fossils and the significance of trichobothria

As part of a wider study into the significance of trichobothrial patterns on scorpion pedipalps, American arachnologist Michael Soleglad and Soviet-born American arachnologist Victor Fet integrated fossil data, especially from the Carboniferous Palaeopisthacanthidae and the Cretcaeous Archaeobuthidae, into their scheme. For the palaeopisthacanthids Soleglad & Fet (2001) were forced to make a composite list, combining data from several species, and conceded that some expected trichobothria on the internal surface of the femur were not visible. For these Carboniferous fossils, a unique trichobothrial arrangement, which they named type ‘P’, was proposed. Although implicitly a buthoid based on its name, Archaeobuthidae was noted for lacking several trichobothria seen in typical buthid type ‘A’ pattern and a novel ‘F1’ pattern for the Cretaceous fossil was proposed. Further details of their hypothesis of trichobothrial evolution can be found Soleglad & Fet (2001), who concluded by recognising a basic phylogeny along the lines of (palaeopisthacanthids (archaeobuthids + all extant scorpions)).

This theme was continued by Soleglad & Fet (2003) as part of a more comprehensive revision of scorpion higher systematics. More generally, Soleglad & Fet (2003) commented on the stocky appearance of the metasoma with all segments of a similar length in many Palaeozoic scorpions as opposed to longer segments in more modern species. The basic pattern of metasomal carinae (*i.e.*, raised lines of tubercles along the segments) was observed to be similar in fossil and living species. Other aspects of fossil morphology such as tarsal shape and spination, as well as cheliceral detention, were briefly discussed. Trichobothrial patterns remained a key part of Soleglad & Fet’s character set, and the ‘P’ and ‘F1’ notations (see above) for palaeopisthacanthids and archaeobuthids were retained. Their final phylogeny was similar to the 2001 study with *Palaeopisthacanthus* and *Archaeobuthus* resolving as the outgroups to Soleglad & Fet’s (2003) four parvorders encompassing all living species: namely Pseudochactida, Buthida, Chaerilida, and Iurida.

We should, however, note the criticisms of South African born, now America-based arachnologist Lorenzo Prendini and American phylogeneticist Ward Wheeler who challenged several aspects of Soleglad & Fet’s (2003) study, from their choice and definition of characters, through to their methods of analysis and its publication in a journal in which the authors are also editors. Specifically for the trichobothria, Prendini & Wheeler (2005) argued that recognising homologous trichobothrial positions remains a contentious issue even among living scorpions. For this reason they cautioned against relying on trichobothrial patterns as diagnostic characters in scorpion higher systematics, and further stated that trichobothria in fossils which cannot be observed may simply be missing for taphonomic reasons and that these setae should be scored as ‘presence only’ data where they can be seen and as unknown (as opposed to being definitively absent) where they are not visible. For further critical discussion of scorpion higher systematics see Prendini & Wheeler (2005), and Fet & Soleglad (2005) for a response.

## New families from the triassic

Two new Triassic scorpions were described by Wilson Lourenço and the French palaeontologist Jean-Claude Gall from the Buntsandstein of France. *Gallioscorpio* Lourenço & Gall, 2004 has a suite of more plesiomorphic characters, including large median eyes on a projection close to the front of the carapace. It was referred to the extinct superfamily Mesophonoidea in its own family Gallioscorpionidae, diagnosed on a combination of features including eye position, a pentagonal sternum, large pectines and a trichobothrial pattern on the pedipalps resembling the ‘A’ configuration.

*Protobuthus* Lourenço & Gall, 2004 is a more gracile, modern looking genus and was assigned to Buthoidea making it potentially the oldest record of a modern superfamily. It was placed in a new, extinct family, Protobuthidae, diagnosed on the slender body, median eyes slightly anterior to the middle of the carapace, the pattern of carinae on the metasoma and again what appears to be an ‘A’ type trichobothrial pattern. Lourenço & Gall (2004) discussed similarities to both Aracheobuthidae and the modern Buthidae, but felt the Triassic fossil could be more of a “proto-element” potentially giving rise to both these lineages. In a wider context, these two fossils from the same locality demonstrate that at least until the Triassic there were scorpion lineages with combinations of plesiomorphic characters living alongside scorpions with modern-looking body plans.

## A cretaceous chaerillid

A more complete scorpion from Burmese amber was described by the American palaeontologist Jorge Santiago-Blay and colleagues. *Electrochaerilus* Santiago-Blay et al., 2004 is the oldest record of the modern Southeast Asian family Chaerilidae which is represented today by a single genus, *Chaerilus* Simon, 1877. At the time of description it was also the oldest scorpion assignable to a modern family. The amber fossil was placed in a new subfamily, Electrochaerilinae, based on differences in cheliceral dentition, the position of trichobothrium V on the pedipalp and the absence of fulcra in the *Electrochaerilus* pectines. Santiago-Blay et al. (2004) discussed the known Cretaceous fossil scorpions, highlighting the fact that Mesozoic taxa may play a crucial role in understanding the development of modern lineages (parvorders) which, according to Soleglad & Fet (2003), may have become established during the Permian to Triassic.

## More baltic amber buthids

Two more extinct genera assigned to Buthidae have been described from Baltic amber. *Palaeoisometrus* Lourenço & Weitschat, 2005b was described as being similar to the modern genus *Isometrus* Ehrenberg, 1828. *Palaeospinobuthus* Lourenço, Henderickx & Weitschat, 2005 was compared to the modern genus *Birulatus* Vachon, 1974, but also showed some similarities to the Cretaceous genus *Archaeobuthus* (see above).

## Akravidae: a subfossil family?

An interesting discovery was the blind chactoid family Akravidae described by Levy (2007) from dead scorpion specimens found in Ayyalon Cave in Israel. Subsequent studies Fet, Soleglad & Zonstein (2011), Fet et al. (2017) have questioned whether a distinct family is justified and have also reported further (dead) specimens from a second cave. Fet, Soleglad & Zonstein (2011) noted that the specimens are not fossilized and are quite well preserved with cuticle which still fluoresces under ultraviolet light as in living scorpions. All of this suggests animals that died fairly recently, but whether it is tens, hundreds or thousands of years ago is difficult to determine. If living examples cannot be found there may be grounds for regarding Akravidae, with its single genus *Akrav* Levy, 2007, as a subfossil group of scorpions.

## Revision of *proscorpius* and its relatives

Dunlop, Tetlie & Prendini (2008) re-examined the American Silurian scorpions from the Bertie Waterlime and concluded that Kjellesvig-Waering’s genera *Archaeophonus* and *Stoermeroscorpio* were probably based on fossils of immature animals and should be treated as synonyms of *Proscorpius*. They assigned *Proscorpius* to Scudder’s (1885) family Proscorpiidae, which was redefined here as scorpions in which all leg coxae, including those of legs I and II, surround and abut a fairly large subtriangular sternum. Based on this sternal character, Dunlop & Jekel (2008) largely followed Jeram (1998) in assigning several other Palaeozoic genera to this family: *Archaeoctonus*, *Hydroscorpius*, *Labriscorpio*, *Pseudoarchaeoctonus* and *Waeringoscorpio*; see also Poschmann et al. (2008) for the same treatment of the latter genus. This renders the families Archaeoctonidae, Hydroscorpiidae, Labriscorpionidae and Waeringoscorpionidae as effective synonyms of Proscorpiidae. The family Stoermeroscorpionidae was also subsumed into Proscorpiidae through the synonymy of its only genus.

## Two extinct buthida families

Two further extinct families in the parvorder Buthida were proposed for mid-Cretaceous scorpions in Burmese amber, although in both cases their superfamilial position is uncertain. Wilson Lourenço and the German amber specialist Alex Beigel described *Chaerilobuthus* Lourenço & Beigel, 2011 in the new family Chaerilobuthidae. It was diagnosed on several features including a trichobothrial pattern between the buthid (‘A’) and chaerilid (‘B’) condition, small bulky pectines and very small spiracles. Lourenço & Beigel (2011) were unable to place the family in either Buthoidea or Chaeriloidea with confidence. Another new genus, *Palaeotrilineatus* Lourenço, 2012, was placed in a second new family, Palaeotrilineatidae. It was diagnosed again on a suite of characters including a large concavity at the front of the carapace, very long pectines and a long aculeus and vesicle making up the telson. Its pedipalp trichobothria were again described as combining elements of the buthid (‘A’), chaerilid (‘B’) and pseudochactid (‘D’) types, thus a new pattern (‘G’) was proposed for this extinct family, which was suggested as having split off at the base of what would now be the three Buthida superfamilies (see Appendix): *i.e.*, Buthoidea, Chaeriloidea and Pseudochactoidea. However, we should note again Prendini & Wheeler’s (2005) critique (see above) of relying too heavily on trichobothrial patterns in scorpion higher systematics.

## Restudy of *paleoscorpius*

The German palaeontologist Gabriele Kühl and colleagues re-examined the Hünsruck scorpion *Paleoscorpius* with a view to testing previous hypotheses about its early branching phylogenetic position and aquatic habitats. Kühl et al. (2012) concluded that there were no convincing morphological characters to support the idea that it lived in water, although it shows no evidence for either coxapophyses or the coxal gnathobases suggested by Jeram (1998). The leg tarsi end in paired claws, with a small dactyl in between the larger ungues, and there are intriguing hints of internal structures revealed by computed tomography for what may be lung-like respiratory organs. They also noted that the Hünsruck Slate does occasionally yield plant fossils, thus it is possible that terrestrial animals might have been preserved here too.

## The first palaeozoic scorpion from africa

The South African palaeontologist Robert Gess described the first fossil scorpion from the Palaeozoic of Africa. *Gondwanascorpio* Gess, 2013 from the Late Devonian of the Eastern Cape in South Africa is incomplete, being known only from a pedipalp and part of the telson. While revealing that scorpions were living in this region of the world at that time, it was placed by Gess (2013) as Scorpiones *incertae sedis*; albeit with comments that the metasomal segments resembled those of mesoscorpions.

## A new silurian genus from canada

A new Silurian genus from Ontario in Canada was described by the Canadian palaeontologist Janet Waddington and colleagues. The familial position of *Eramoscorpius* Waddington, Rudkin & Dunlop, 2015 was left open, but the authors did discuss its possible significance for having walking legs with a short tarsus which thus appear to be approaching the condition present in the legs of modern scorpions. Further morphological details for the genus were later added by Haug, Wagner & Haug (2019).

## Further records from burmese amber

Burmese amber yielded a new genus, *Archaeoscorpiops* Lourenço, 2015a, based on a pedipalp claw and assigned to a new subfamily of the previously described extinct family Palaeoeuscorpiidae. *Burmesescorpiops* Lourenço, 2016b was described as palaeoeuscorpiid too. Palaeoburmesebuthinae Lourenço (2015b) was proposed as a subfamily of the extinct family Archaeobuthidae. It was based on a suite of characters and subsequently raised to full family status by Lourenço (2016a). A further article added a new genus, *Betaburmesebuthus Lourenço in* Lourenço & Beigel (2015), from the same subfamily. A more general overview of fossil scorpions was published by Lourenço (2015c). The Italian scorpion specialist Andrea Rossi described another family and genus from Burmese amber. *Sucinlourencous* Rossi, 2015 was assigned to the new, extinct family Sucinlourencoidae Rossi (2015). It was defined on a range of characters, the most important of which was a trichobothrial pattern with five ventral trichobothria on the pedipalp chelae–note again Prendini & Wheeler’s (2005) critiques of diagnosing taxa on trichobothria alluded to above–at least five external trichobothria and one internal trichobothrium in an unusual position; legs III and IV lack tarsal spurs. It was not formally assigned to a superfamily, but Rossi (2015) considered it most closely related to Palaeotrilineatidae which currently occupies an uncertain position within the parvorder Buthida. Sucinlourencoidae is thus provisionally placed in a similar position here (see Appendix). Another genus Burmese amber genus, *Archaeoananteroides* Lourenço *in* Lourenço & Velten (2016) was described in Buthidae.

## A possible euscorpiid for italy

Shale-preserved fossil scorpions from the Cenozoic are not as common as amber ones, thus *Eoeuscorpius* Kühl & Lourenço, 2017 is an interesting addition. This rather squat fossil with robust pedipalps from the Pesciara Lagerstätte of Italy was tentatively referred by Kühl & Lourenço (2017) to the living family Euscorpiidae.

## New permian scorpions

Martin Dammann from Germany described a new genus of fossil scorpion from the Early Permian of the Ural region (“Angara-Land”) of Russia. *Permomatveevia* Dammann, 2017 was compared to the Carboniferous genus *Eoscorpius*, but was not placed in a family. Dammann’s (2017) article is, in places, erroneous–he referred to scorpions throughout as insects–but the published photographs confirm that it was a fairly gracile, long-legged scorpion with median eyes towards the front of the carapace.

Another Permian scorpion was described by the Brazilian palaeontologist Ariel Milani Martine and colleagues. *Suraju* Martine et al., 2020 is an important discovery, being the first Palaeozoic scorpion from South America. It comes from the Teresina Formation in the Santa Catarina region of Brazil and was assigned to an uncertain familial position as Orthosternina *incertae sedis*.

## Recent developments

Further new taxa continue to be described from Burmese amber. *Cretaceoushormiops* Lourenço, 2018 and *Cretaceousopisthacanthus* Lourenço *in* Lourenço & Velten (2021) were both placed in Protoischnuridae; here treated as a valid family (see above). *Cretaceousbuthus* Lourenço *in* Lourenço & Velten (2022) is of note for having been tentatively assigned to Buthidae; see also Lourenço & Velten (2023). If this interpretation is correct this genus would be the oldest record of the modern family. Xuan, Cai & Huang (2023a) suggested that the Burmese amber genus *Spinoburmesebuthus* could be a junior synonym of *Betaburmesebuthus*. Also in Burmese amber, Xuan, Cai & Huang (2023b) described some juvenile Chaerilidae and suggested that they belong to the living Southeast Asian genus *Chaerilus*. If this assignment is correct, it would be the oldest material belonging to a modern genus.

The Swiss geologist Fabio Magnani and colleagues described a well-preserved shale fossil from the Triassic of Monte San Giorgio in Switzerland. *Protochactas* Lourenço, Magnani & Stockar *in* Magnani, Stockar & Lourenço (2022) was placed in a new, extinct chactoid family, Protochactidae, diagnosed on a suite of characters differentiating it from other fossil and living chactoids. It represents the oldest putative record of the superfamily Chactoidea and of the wider parvorder Iurida. Another Triassic fossil from the same locality was described by Viaretti, Bindellini & Dal Sasso (2023) and placed in the previously described family Protobuthidae (see above).

As a prelude to the present study, Dunlop & Garwood (2023) re-examined the nomenclature of the Silurian genus *Palaeophonus*. Two fossils assigned to this genus were treated as *nomina dubia* on account of their incompleteness and/or lack of scorpion features. *Allopalaeophonus* was formally synonymised with *Palaeophonus*, which renders Allopalaeophonide a synonym of Palaeophonidae; something anticipated by Stockwell (1989) and Jeram (1998), who both noted that the fossils on which these genera were based are very similar.

More recently in Burmese amber, the new genus *Paranotaburmesebuthus* Lourenço *in* Lourenço & Velten (2024) was placed in the extinct family Palaeoburmesebuthidae. Finally, Ythier (2024) proposed revalidating the modern family Ananteridae (previously part of Buthidae), and further suggested that the fossil genera *Archaeoananteroides* from Burmese amber and *Palaeoananteris* and *Palaeotityobuthus* (both from Baltic amber) belong here too. Baltic examples would be slightly surprising from a biogeographical perspective as the modern members of this family are predominantly South American, albeit with some African and Asian species.

## Discussion

### Scorpion evolution

**Figure 9:**
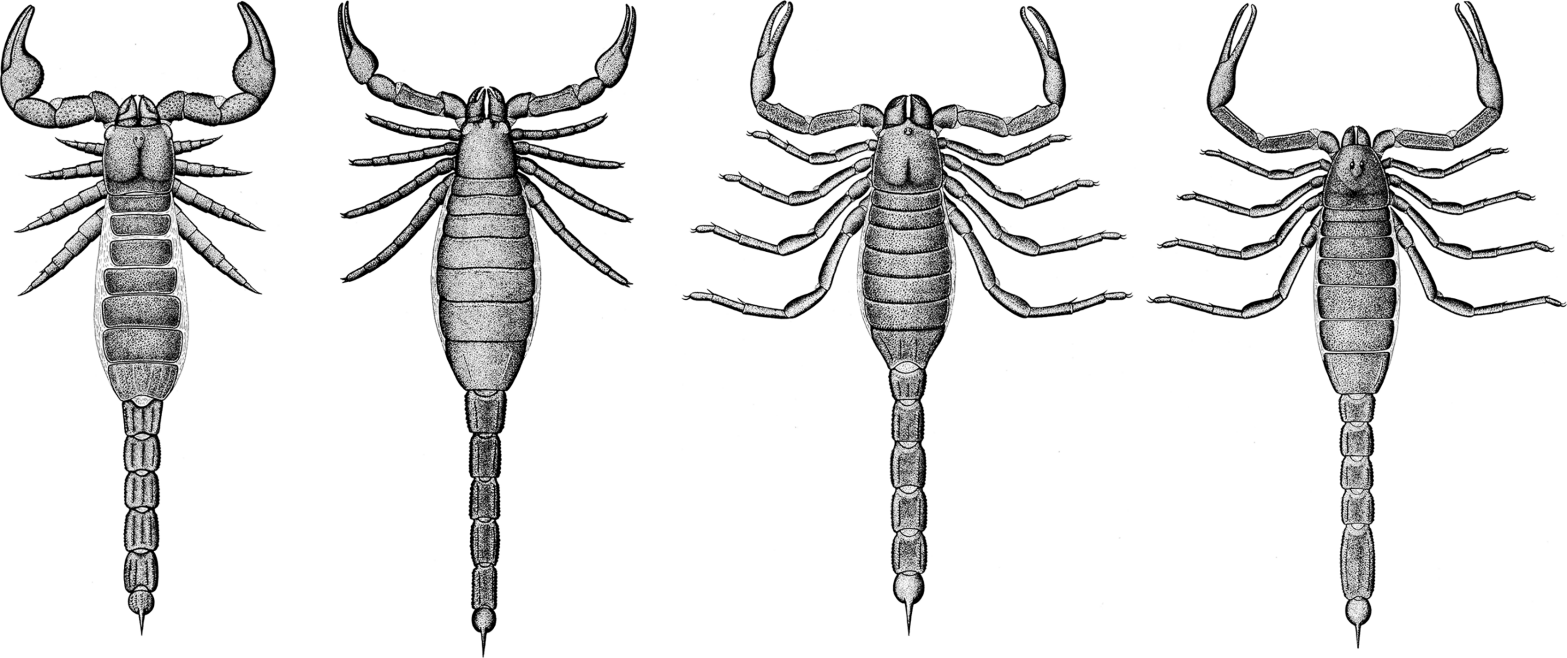
Reconstructions of key fossil scorpion genera. From left to right are shown: the Silurian *Palaeophonus*, of note are leg proportions and digitigrade stance; Silurian *Proscorpius*, the carapace of which bore both median and compound lateral eyes; Lower Carboniferous *Pulmonoscorpius*, which for which there is unequivocal evidence of book lungs; and Upper Carboniferous *Compsosorpius*, which is an early representative of the orthosternous scorpions.

This review highlights the rich fossil record of the Scorpiones (Figs. 2, 3), which stretches back to the Silurian (Fig. 1), and charts a suite of notable morphological changes as crown group scorpions evolve. Early members of the clade had pointed, anteriorly-shorter legs, with a tarsus and basitarsus broadly equivalent in length, or with the former longer than the latter (Fig. 9). This is suggestive of a digitigrade stance, *i.e.*, that the animals locomoted on the tips of its walking limbs, in contrast to a plantigrade stance—as seen in orthostern scorpions—facilitated by a shorter tarsus than basitarsus. Alongside this shift in distal limb morphology, there are modifications to the proximal limbs associated with the evolution of the stomotheca feeding apparatus (a preoral tube comprising, in part, coxapophyses of the palps and first two walking limbs), which was present in Upper Carboniferous orthosterns (Legg et al., 2012), but not present in early representatives of the group (Dunlop, Tetlie & Prendini, 2008). The evolution of crown group Scorpiones also sees a marked posterior-ward shift in the median eyes, from an anterior position to one midway along the dorso-ventral axis of the carapace, and the loss of compound lateral eyes. These patterns are important for understanding scorpion origins, and have been the basis for debate regarding the early ecology of the group, and thus the timing and process of terrestiralisation within the scorpions and/or arachnids more broadly. However, a more detailed picture will presumably be possible with an updated phylogeny for the group.

### Scorpion taxonomy

**Figure 10:**
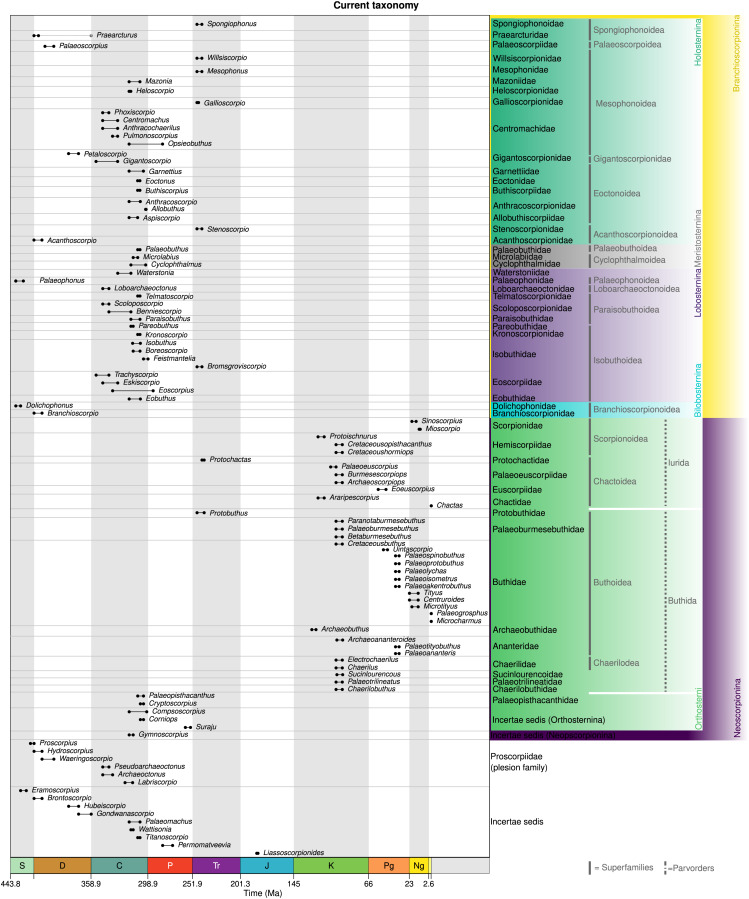
The current status of fossil scorpion taxonomy, which represents a combination of numerous schemes. The scheme of Kjellesvig-Waering (1986) has been elaborated on and modified since publication in a piecemeal fashion. Given its complexity, in parts of the more recent literature authors have tended to use informal groupings rather than the taxonomy presented above.

What is evident from this review is the rather chaotic early phases of research in the late 19^th^ and early 20^th^ century in which several family groups were named, only to be ignored or abandoned in later studies usually without any formal discussion or synonymy. The result is that the taxonomy of fossil scorpions is complex (Fig. 10), and-given the origins of the arachnologists introduced in the historical overview—has a bias towards localities in the Northern Hemisphere. Indeed much of the previous work on fossil scorpions has been concentrated into the hands of a relatively small number of individuals (primarily Leonard Wills, Reginald Pocock, Alexander Petrunkevitch, Erik Kjellesvig-Waering, Andrew Jeram and Wilson Lourenço) and we hope that future data from a more diverse set of both localities and authors will lead to a better understanding of evolutionary pathways. The complex nature of extinct scorpions’ higher systematics also results from past hypotheses of relationships that often relied solely on configurations of the coxo-sternal region as diagnostic characters, making it difficult to test the position of fossils where only the dorsal surface is preserved. Furthermore, Petrunkevitch’s (1955) use of characters which were equivocal in several key fossils, *e.g.*, “Tarsi not preserved, but presumably ending in a pair of claws”, is also unhelpful.

As such, the current status represents a combination of different schemes, often modified in a piecemeal fashion in subsequent publications. Most of the historical family names for Palaeozoic and Mesozoic species were eventually adopted by Kjellesvig-Waering (1986), in addition to the new names he proposed himself (Fig. 6). However, as other authors have commented (*e.g.*, Dunlop, Tetlie & Prendini, 2008), Kjellesvig-Waering’s monograph is problematic both in terms of the high number of mutually exclusive taxa and in the reliability of the morphological data underlying his major groups. Similar-looking scorpions were placed in different infraorders based on the supposed condition of the ventral mesosomal plates; for example Dunlop & Garwood (2023) recently synonymised the holostern *Allopaleophonus* with the lobostern *Palaeophonus*. Identical coxo-sternal morphologies also ended up split across several different groups, implying either much convergent evolution or, more likely, a series of non-monophyletic taxa. Despite these problems, the current classification of fossil scorpions (*e.g.*, Dupre, 2024; see also Appendix, Fig. 10) remains a hybrid between Kjellesvig-Waering’s names (itself incorporating a handful of previously proposed groupings), and the hypotheses proposed in the cladistic analyses of Stockwell (1989) and Jeram (1994a, 1994b, 1998). Some progress towards revising families and genera has begun (Dunlop, Tetlie & Prendini, 2008; Legg et al., 2012; Dunlop & Garwood, 2023) which should help reduce the number of superfluous higher taxa. Nevertheless, it is clear that the fossil record of the Scorpiones is in need of systematic restudy and a thorough taxonomic revision, rationalising the current scheme.

### Current taxonomic divisions and future directions

Ideally future research directions should include a cladistic analysis incorporating fossil species: a first step towards this is to complement the fossil species list included herein with a list of diagnostic characters, that might form the basis of future phylogenetic studies. Integrating all the published family and genus names into a single, final phylogeny will not be possible because so many of them derive from incomplete material. For example, at least four Palaeozoic genera (*Brontoscorpio*, *Palaeomachus, Titanoscorpio* and *Waterstonia*) were based on fossils consisting of an isolated pedipalp claw or finger. As such, a valuable approach to the study of fossil scorpions in future would be to create a morphological cladistic matrix. Ideally, this should: include all key anatomical characters; incorporate members of major extant clades; contain a broad selection of carefully selected fossil scorpion taxa, with a focus on those that are either completely preserved, or are thought to occupy an important position in terms of the evolutionary transitions within scorpions (we note that missing data is not inherently problematic in cladistic studies: Wiens, 1998); and also encompass important outgroups, particularly fossil aquatic chelicerates. Ultimately, this could then be included in a total evidence analysis that allows this morphology to be combined with molecular characters creating a data rich phylogeny (*i.e.*, with multiple partitions) incorporating extinct taxa. This would be a highly valuable undertaking, in addition to providing a basis for a revised taxonomy, given that fossils are key to understanding the timing of key transitions (Mongiardino Koch, Garwood & Parry, 2023), the rates of evolution (Slater & Harmon, 2013), and improve the accuracy of phylogenetic inference (Mongiardino Koch, Garwood & Parry, 2021). In the remainder of our discussion, we thus try to clarify relevant aspects of the current taxonomy that would assist with both a taxonomic revision, and the construction of a phylogeny.

**Figure 11:**
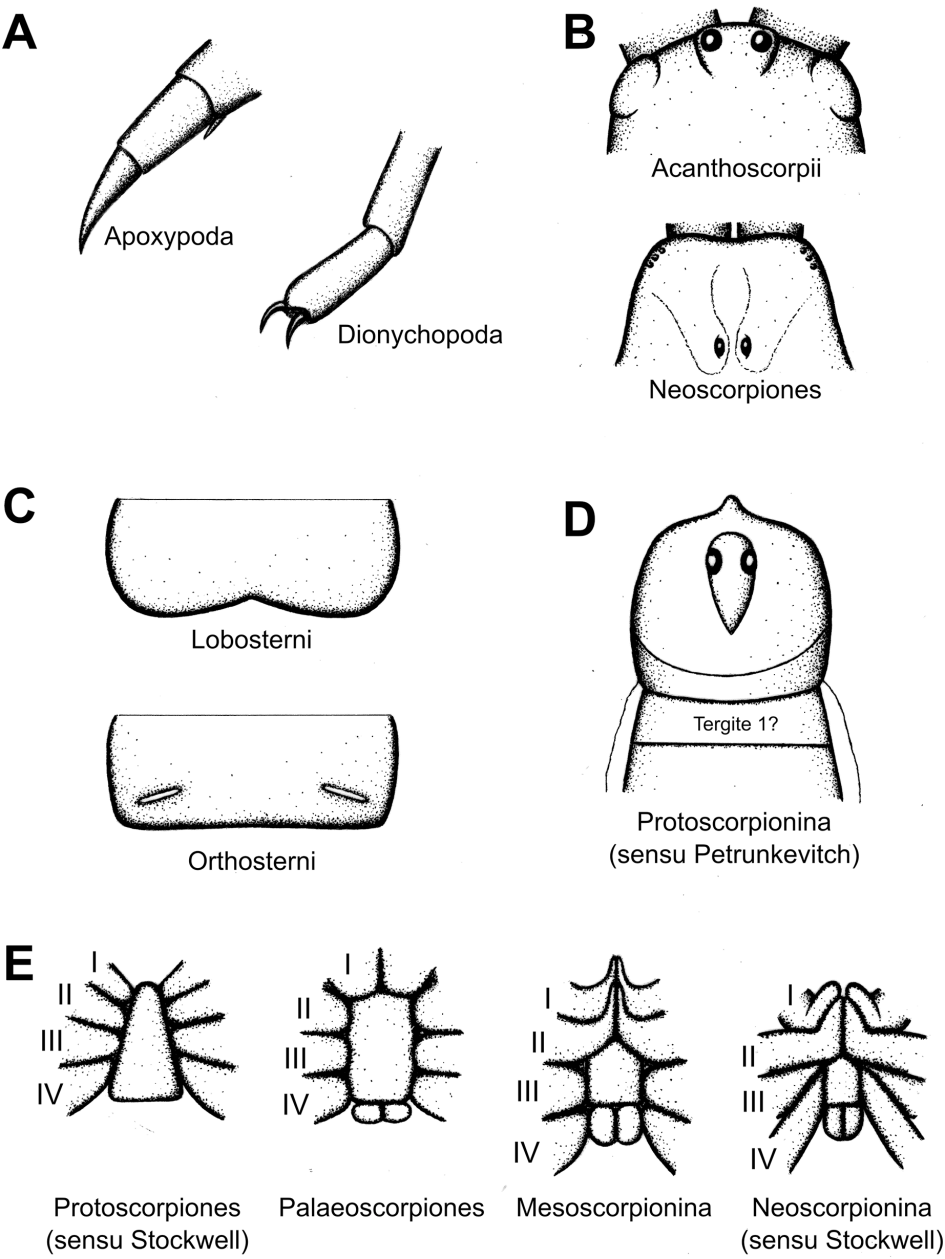
Key morphological characters used to define fossil scorpion groups that can be found in the literature. See text for discussion.

In terms of higher clade names, the following possible divisions of the scorpions present themselves from the historical literature. From Thorell & Lindström (1885; Fig. 4), Apoxypoda is an available name for scorpions with a single pointed tarsus and Dionychopoda is an available name for scorpions with paired tarsal claws (Fig. 11A). From Scudder (1885) Anthacoscorpii is an available name for scorpions with median eyes in an anterior position (Fig. 11B), although it should be cautioned that it has been used historically for different ranks ranging from a family to a suborder. Neoscorpiones is an available name for scorpions with median eyes in a more posterior position. From Pocock (1911; Fig. 6) Lobosterni is an available name for scorpions with bilobed mesosomal ventral plates and Orthosterni is an available name for scorpions with a modern configuration of the ventral mesosomal sternites with spiracles opening in the middle of the plate (Fig. 11C). From Petrunkevitch (1949) Protoscorpionina is an available name for scorpions which, in its original usage, retained the first opisthosomal tergite (Fig. 11D), but which was redefined by Stockwell (1989) as scorpions with stubby legs and all coxae surrounding the sternum. Petrunkevitch’s Euscorpionina is an available name for scorpions in which the putative first opisthosomal tergite has been lost. Kjellesvig-Waering’s (1986; Fig. 6) Holosternina, Meristosternina and Bilobosternina are available names for scorpions with alternative patterns of ventral plate morphology (Fig. 11E). Stockwell’s Palaeoscorpiones is an available name for scorpions with longer legs and paired claws, but only the first pair of leg coxae meeting in front of the mouth. His Mesoscorpionina is an available name for scorpions where coxae IV abut the genital operculum, multifaceted lateral eyes are retained and the median eyes are on an anterior carapace projection. His Neoscorpionina has coxae IV abutting the sternum (not the genital operculum) a reduced number of lenses in the lateral eyes and posterior displacement of the median eyes.

These combinations also suggest a suite of key morphological characters which are likely to be most informative for higher systematics. They include the length of the legs and the shape of the tarsus and its claws, the coxosternal region and especially the presence/absence of coxapophyses, the presence/absence of pectines, the sclerites (and any spiracles) on the underside of the mesosoma, the number of lateral eye lenses and the position of the median eyes. A caveat here is that (a) some of these morphologies, like leg length and median eye position, are gradations rather than simple presence/absence characters and (b) phylogenetic analysis may be needed to differentiate between plesiomorphic and apomorphic character states, and thus whether these names are likely to be defining monophyletic groups. A key challenge in this regard, alongside the incomplete fossils mentioned previously, is choosing an appropriate outgroup, given the current lack of resolution for arachnids outside the Pantetrapulmonata; see Giribet (2018, fig. 2) or Sharma, Ballesteros & Santibáñez-López (2021, fig. 1) for a summary of alternative positions for scorpions with respect to other arachnids. It is also clear that some of the putative groupings we have outlined will overlap. For example the two-clawed Dionychopoda group includes both ‘lobostern’ fossils and crown-group scorpions and their relatives with the modern orthostern condition. Some names probably have little merit, being based on questionable characters. Protoscorpionina *sensu* Petrunkevitch is a case in point, as we are not aware of any fossil scorpion which convincingly expresses a retained first tergite. Bilobosternina is another group where the only evidence for its key diagnostic character is a drawing by Kjellesvig-Waering (1986, fig. 101); even the compilers of his monograph had to add several footnotes querying his interpretations.

## Conclusions

In summary (see also Appendix; Fig. 10), only Thorell & Lindström’s Neoscorpiones and Pocock’s Orthosterni remain in regular use in the systematic/phylogenetic literature (*e.g.*, Soleglad & Fet, 2003; Santiago-Blay et al., 2004; Baptista et al., 2006) where they are usually treated as a suborder and infraorder respectively. Neoscorpions are usually now defined as those scorpions in which the number of lateral eye lenses has been reduced and the median eyes have shifted back towards the middle of the carapace. A difficulty here is that while many Palaeozoic and some Mesozoic fossils are presumed to have had semi-compound multi-faceted eyes, only a few are preserved well-enough to show this unequivocally (*e.g.*, Wills, 1910; Miether & Dunlop, 2016). Orthosterns are explicitly defined as those scorpions in which the spiracle openings are located within the sternite and include some fossil and all living species. Scorpions with this morphology first appear in the Late Carboniferous (Vogel & Durden, 1966; Jeram, 1994b) although, as with coxosternal morphology, this character can only be tested in fossils preserving the ventral surface of the opisthosoma with high fidelity.

We suggest that there are now two major challenges in fossil scorpion systematics. First, we need to build on the work of Stockwell (1989) and Jeram (1994a, 1994b, 1998) towards reconstructing and refining the largely Paleozoic stem-group leading up to the infraorder Orthosterni. Possible pathways of morphological evolution in early scorpions were investigated by Haug, Wagner & Haug (2019), who identified shifts in the sternal, genital and pectine regions in particular, such as the incorporation of additional limb coxae into the feeding apparatus (as coxapophyses) and the possible evolution of the pectines within the early scorpions from a lamellate respiratory organ. These potentially apomorphic character states still need to be integrated into a formal systematic framework. Specifically, fruitful next steps could incorporate: 1) Complementing the species list included herein with a diagnostic character list for each of these taxa; 2) Building from this a character- and taxon-rich morphological cladistic analysis incorporating representatives of living groups, a broad selection of the most completely preserved fossil taxa, and a sample of key fossil outgroups including eurypterids, synziphosurines and xiphosurans; 3) A total evidence analysis combining the morphology of key fossil taxa within a molecular framework for extant species; and 4) A wholescale revision of the higher taxonomy of the group either informed by, or as a prelude to, the proposed phylogenetic studies.

Second, within the crown-group relationships between the eight extinct orthostern families from the Mesozoic and the living family groups should be explored. Are they early branches of the entire Orthosterni, early representatives of either the parvorder Buthida or Iurida, extinct sister-groups of modern families, or a combination of these? How useful are trichobothrial patterns on the pedipalps for resolving relationships between extinct and living families? Baptista et al. (2006) cautioned against using names for fossil families based on living family groups unless there is convincing evidence that the animals are closely related. Such hypotheses should, where possible, be based not merely on general resemblances, but should ideally be more firmly grounded by expressing them in terms of shared apomorphic character states.

## Supplemental Information

10.7717/peerj.18557/supp-1 Supplemental Information 1 Data and Script. The PBDB scorpion data, a standalone R script to conduct analyses and create outputs, as bash script to run this R script, a read me, and Figure S1. The readme contains full instructions.

10.7717/peerj.18557/supp-2 Supplemental Information 2 A review of fossil scorpion higher systematics - Appendix - Taxonomic Summary. Summary classification of fossil scorpions down to genus level based on Dunlop & Garwood, 2023. There are currently 76 extinct genera and 43 extinct families in the fossil record, with eight living families and five living genera also known as fossils.

## Additional Information and Declarations

### Data Availability

The following information was supplied regarding data availability: The raw data is available in the Supplemental File.
