# Supplementary material for: A review of fossil scorpion higher systematics
Source: PeerJ. 2024 Dec 6;12:e18557. doi: 10.7717/peerj.18557 (PMC11627080; doi:10.7717/peerj.18557)
Supplement: Supplemental Information 1 — The PBDB scorpion data, a standalone R script to conduct analyses and create outputs, as bash script to run this R script, a read me, and Figure S1. The readme contains full instructions. [file peerj-12-18557-s001.zip › SI_Figure_01.pdf]

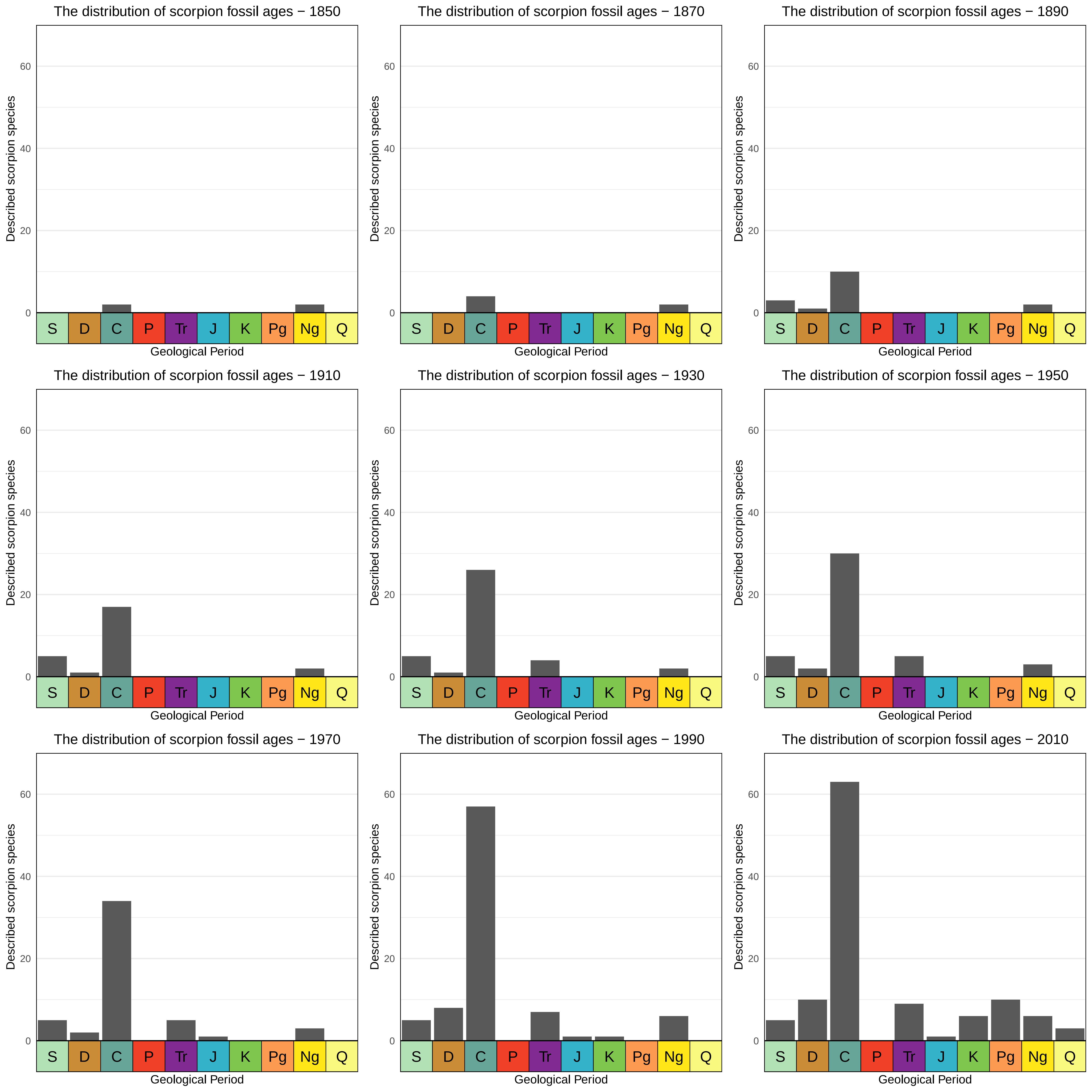
The distribution of scorpion fossil ages − 1850
The distribution of scorpion fossil ages − 1870
The distribution of scorpion fossil ages − 1890
The distribution of scorpion fossil ages − 1910
The distribution of scorpion fossil ages − 1930
The distribution of scorpion fossil ages − 1950
The distribution of scorpion fossil ages − 1970
The distribution of scorpion fossil ages − 1990
The distribution of scorpion fossil ages − 2010
Described scorpion species
Geological Period
0
20
40
60
S
D
C
P
Tr
J
K
Pg
Ng
Q
