# Supplementary material for: A review of fossil scorpion higher systematics
Source: PeerJ. 2024 Dec 6;12:e18557. doi: 10.7717/peerj.18557 (PMC11627080; doi:10.7717/peerj.18557)
Supplement: Supplemental Information 2 — Summary classification of fossil scorpions down to genus level based on Dunlop & Garwood, 2023. There are currently 76 extinct genera and 43 extinct families in the fossil record, with eight living families and five living genera also known as fossils. [file peerj-12-18557-s002.docx]

A review of fossil scorpion higher systematics - Appendix

**Jason A. Dunlop^1^ & Russell J. Garwood^2^**

^1^Museum für Naturkunde, Leibniz Institute for Evolution and Biodiversity Science Invalidenstrasse 43, D-10115 Berlin, Germany; E-mail: jason.dunlop@mfn.berlin

^2^Department of Earth and Environmental Science, The University of Manchester, Manchester, UK; Natural History Museum, London, UK. E-mail: russell.garwood@manchester.ac.uk

Summary classification of fossil scorpions down to genus level based on Dunlop et al. (2023). There are currently 76 extinct genera and 43 extinct families in the fossil record, with eight living families and five living genera also known as fossils.

SCORPIONES C. L. Koch, 1851

† Proscorpiidae Scudder, 1885 (plesion family)

*=* † Archaeoctonidae Petrunkevitch, 1949

*=* † Hydroscorpionidae Kjellesvig-Waering, 1986

*=* † Labriscorpionidae Kjellesvig-Waering, 1986

= † Stoermeroscorpioniidae Kjellesvig-Waering, 1986

= † Waeringoscorpionidae Størmer, 1970

† *Archaeoctonus* Pocock, 1911 (Carboniferous: 1 species)

† *Hydroscorpius* Kjellesvig-Waering, 1986 (Devonian: 1 species)

† *Labriscorpio* Leary, 1980 (Carboniferous: 1 species)

† *Proscorpius* Whitfield, 1885*b* (Silurian: 1 species)

*=* † *Archaeophonus* Kjellesvig-Waering, 1966*b*

= † *Stoermeroscorpio* Kjellesvig-Waering, 1986

† *Pseudoarchaeoctonus* Kjellesvig-Waering, 1986 (Carboniferous: 1 species)

† *Waeringoscorpio* Størmer, 1970 (Devonian: 1 species)

† BILOBOSTERNINA Kjellesvig-Waering

† Branchioscorpionoidea Kjellesvig-Waering, 1986

† Branchioscorpionidae Kjellesvig-Waering, 1986

† *Branchioscorpio* Kjellesvig-Waering, 1986 (Devonian: 1 species)

† Dolichophonidae Petrunkevitch, 1953

† *Dolichophonus* Petrunkevitch, 1949 (Silurian: 1 species)

† HOLOSTERNINA Kjellesvig-Waering, 1986

† Acanthoscorpionoidea Kjellesvig-Waering, 1986

† Acanthoscorpionidae Kjellesvig-Waering, 1986

† *Acanthcoscorpio* Kjellesvig-Waering, 1986 (Devonian: 1 species)

† Stenoscorpionidae Kjellesvig-Waering, 1986

† *Stenoscorpio* Kjellesvig-Waering, 1986 (Triassic: 2 species)

† Eoctonoidea Kjellesvig-Waering, 1986

† Allobuthiscorpiidae Kjellesvig-Waering, 1986

*Allobuthiscorpius* is now a junior synonym (see below)

† *Aspiscorpio* Kjellesvig-Waering, 1986 (Carboniferous: 2 species)

† Anthracoscorpionidae Frič, 1904

† *Allobuthus* Kjellesvig-Waering, 1986 (Carboniferous: 1 species)

† *Anthracoscorpio* Kušta, 1885 (Carboniferous: 2 species)

† Buthiscorpiidae Kjellesvig-Waering, 1986

† *Buthiscorpius* Petrunkevitch, 1953 (Carboniferous: 1 species)

† Eoctonidae Kjellesvig-Waering, 1986

† *Eoctonus* Petrunkevitch, 1913 (Carboniferous: 1 species)

† Garnettiidae Dubinin, 1962

† *Garnettius* Petrunkevitch, 1953 (Carboniferous: 1 species)

† Gigantoscorpionoidea Kjellesvig-Waering, 1986

† Gigantoscorpionidae Kjellesvig-Waering, 1986

= † Petaloscorpionidae Kjellesvig-Waering, 1986

† *Gigantoscorpio* Størmer, 1963 (Carboniferous: 1 species)

† *Petaloscorpio* Kjellesvig-Waering, 1986 (Devonian: 1 species)

† Mesophonoidea Wills, 1910

† Centromachidae Petrunkevitch, 1953

= † Anthracocherilidae Kjellesvig-Waering, 1986

= † Opsieobuthidae Kjellesvig-Waering, 1986

= † Phoxiscorpionidae Kjellesvig-Waering, 1986

† *Anthracochaerilus* Kjellesvig-Waering, 1986 (Carboniferous: 1 species)

† *Centromachus* Thorell & Lindström, 1885 (Carboniferous: 1 species)

† *Opsieobuthus* Kjellesvig-Waering, 1986 (Carboniferous–Permian: 2 species)

† *Phoxiscorpio* Kjellesvig-Waering, 1986 (Carboniferous: 1 species)

† *Pulmonoscorpius* Jeram, 1994*a* (Carboniferous: 1 species)

† Gallioscorpionidae Lourenço & Gall, 2004

† *Gallioscorpio* Lourenço & Gall, 2004 (Triassic: 1 species)

† Heloscorpionidae Kjellesvig-Waering, 1986

† *Heloscorpio* Kjellesvig-Waering, 1986 (Carboniferous: 1 species)

† Mazoniidae Petrunkevitch, 1913

† *Mazonia* Meek & Worthen, 1868*b* (Carboniferous: 1 species)

† Mesophonidae Wills, 1910

† *Mesophonus* Wills, 1910 (Triassic: 3 species)

† Willsiscorpionidae Kjellesvig-Waering, 1986

† *Willsiscorpio* Kjellesvig-Waering, 1986 (Triassic: 1 species)

† Palaeoscorpoidea Lehmann, 1944

† Palaeoscorpionidae Petreunkevitch, 1953

† *Palaeoscorpio* Lehmann, 1944 (Devonian: 1 species)

Kühl *et al*. (2012) simply listed the genus unplaced under Protoscorpionina

† Spongiophonoidea Kjellesvig-Waering, 1986

† Praearcturidae Kjellesvig-Waering, 1986

† *Praearcturus* Woodward, 1871*a* (Devonian: 1 species)

† Spongiophonidae Kjellesvig-Waering, 1986

† *Spongiophonus* Wills, 1947 (Triassic: 1 species)

† MERISTOSTERNINA Kjellesvig-Waering, 1986

† Cyclophthalmoidea Thorell & Lindström, 1885

† Cyclophthalmidae Thorell & Lindström, 1885

† *Cyclophthalmus* Corda, 1835 (Carboniferous: 3 species)

† Microlabiidae Kjellesvig-Waering, 1986

† *Microlabis* Corda, 1839 (Carboniferous; 1 species)

† Palaeobuthoidea Kjellesvig-Waering, 1986

† Palaeobuthidae Kjellesvig-Waering, 1986

† *Palaeobuthus* Petrunkevitch, 1913 (Carboniferous: 1 species)

= † *Mazoniscorpio* Wills, 1960

† LOBOSTERNINA Pocock, 1911.

† Isobuthoidea Petrunkevitch, 1913

† Eobuthidae Kjellesvig-Waering, 1986

† *Eobuthus* Frič, 1904 (Carboniferous: 3 species)

† Eoscorpiidae Scudder, 1884

† *Eoscorpius* Meek & Worthen, 1868*a* (Carboniferous- Permian: 7 species)

= † *Alloscorpius* Petrunkevitch, 1949

= † *Europhthalmus* Petrunkevitch, 1949

= † *Lichnophthalmus* Petrunkevitch, 1949

= † *Trigonoscorpio* Petrunkevitch, 1913

= † *Typhloscorpius* Petrunkevitch, 1949

† *Eskiscorpio* Kjellesvig-Waering, 1986 (Carboniferous: 1 species)

† *Trachyscorpio* Kjellesvig-Waering, 1986 (Carboniferous: 1 species)

† Isobuthidae Petrunkevitch, 1913

† *Boreoscorpio* Kjellesvig-Waering, 1986 (Carboniferous: 1 species)

† *Bromsgroviscorpio* Kjellesvig-Waering, 1986 (Triassic: 1 species)

† *Feistmantelia* Frič, 1904 (Carboniferous: 1 species)

† *Isobuthus* Frič, 1904 (Carboniferous: 2 species)

† Kronoscorpionidae Kjellesvig-Waering, 1986

† *Kronoscorpio* Kjellesvig-Waering, 1986 (Carboniferous: 1 species)

† Pareobuthidae Kjellesvig-Waering, 1986

† *Pareobuthus* Wills, 1959 (Carboniferous: 1 species)

† Paraisobuthoidea Kjellesvig-Waering, 1986

† Paraisobuthidae Kjellesvig-Waering, 1986

† *Paraisobuthus* Kjellesvig-Waering, 1986 (Carboniferous: 4 species)

† Scoloposcorpionidae Kjellesvig-Waering, 1986

† *Benniescorpio* Wills, 1960 (Carboniferous: 1 species)

† *Scoloposcorpio* Kjellesvig-Waering, 1986 (Carboniferous: 1 species)

† Telmatoscorpionidae Kjellesvig-Waering, 1986

† *Telmatoscorpio* Kjellesvig-Waering, 1986 (Carboniferous: 1 species)

† Loboarchaeoctonoidea Kjellesvig-Waering, 1986

† Loboarchaeoctonidae Kjellesvig-Waering, 1986

† *Loboarchaeoctonus* Kjellesvig-Waering, 1986 (Carboniferous: 1 species)

† Palaeophonoidea Thorell & Lindström, 1884

† Palaeophonidae Thorell & Lindström, 1884

= † Allopalaeophonidae Kjellesvig-Waering, 1986

† *Palaeophonus* Thorell & Lindström, 1884 (Silurian: 2 species)

= † *Allopalaeophonus* Kjellesvig-Waering, 1986

superfamily uncertain

† Waterstoniidae Kjellesvig-Waering, 1986

† *Waterstonia* Kjellesvig-Waering, 1986 (Carboniferous: 1 species)

NEOSCORPIONINA Thorell & Lindström, 1885 (suborder)

Neoscorpionina *incertae sedis*

† *Gymnoscorpius* Jeram, 1994*b* (Carboniferous: 1 species)

ORTHOSTERNI Pocock, 1911 (infraorder)

Orthosternina *incertae sedis*

† *Compsoscorpius* Petrunkevitch 1949 (Carboniferous: 1 species)

= † *Allobuthiscorpius* Kjellesvig-Waering, 1986

= † *Coseleyscorpio* Kjellesvig-Waering, 1986

= † *Leioscorpio* Kjellesvig-Waering, 1986

= † *Lichnoscorpius* Petrunkevitch, 1949

= † *Pseudobuthiscorpius* Kjellesvig-Waering, 1986

= † *Typhlopisthacanthus* Petrunkevitch, 1949

† *Corniops* Jeram, 1994*b* (Carboniferous: 1 species)

† *Suraju* Martine, Ricardi-Branco, Beloto & Jurigan, 2020 (Permian: 1 species)

† Palaeopisthacanthidae Kjellesvig-Waering, 1986

Legg *et al*. (2012) excluded *Composcorpius* from this family as its inclusion made it paraphyletic in Jeram’s (1994) cladogram

† *Cryptoscorpius* Jeram, 1994*b* (Carboniferous: 1 species)

† *Palaeopisthacanthus* Petrunkevitch, 1913 (Carboniferous: 2 species)

BUTHIDA Soleglad & Fet 2003 (parvorder)

superfamily uncertain

† Chaerilobuthidae Lourenço & Beigel, 2011

† *Chaerilobuthus* Lourenço & Beigel, 2011 (Cretaceous: 10 species)

† Palaeotrilineatidae Lourenço, 2012*b*

† *Palaeotrilineatus* Lourenço, 2012*b* (Cretcaeous: 1 species)

† Sucinlourencoidae Rossi, 2015

† *Sucinlourencous* Rossi, 2015 (Cretaceous: 1 species)

Pseudochactoidea Gromov, 1998

Pseudochactidae Gromov, 1998 (Recent)

Chaerilodea Pocock, 1893

Chaerilidae Pocock, 1893

† *Electrochaerilus* Santiago-Blay *et al*., 2004 (Cretaceous: 2 species)

*Chaerilus* Simon, 1877 (Cretaceous – Recent: 1 fossil species)

Buthoidea C. L. Koch, 1837

† Protobuthidae Lourenço & Gall, 2004

† *Protobuthus* Lourenço & Gall, 2004 (Triassic: 1 species)

† Archaeobuthidae Lourenço, 2001

† *Archaeobuthus* Lourenço, 2001 (Cretaceous: 1 species)

† Palaeoburmesebuthidae Lourenço, 2015*b*

† *Betaburmesebuthus Lourenço in* Lourenço & Beigel, 2015 (Cretaceous: 7 species)

† *Palaeoburmesebuthus* Lourenço, 2002 (Cretcaeous: 4 species)

† *Spinoburmesebuthus* Lourenço, 2002 (Cretcaeous: 2 species)

Buthidae C. L. Koch, 1837

*Centruroides* Marx, 1890*a* (Neogene–Recent : 1 fossil species)

† *Cretaceousbuthus* Lourenço *in* Lourenço & Velten, 2022*b* (Cretaceous: 1 species)

tentative assignment to Buthidae

*Microcharmus* Lourenço, 1995 (Quaternary – Recent: 1 subfossil species)

*Microtityus* Kjellesvig-Waering, 1966*c* (Neogene – Recent)

† *Palaeoakentrobuthus* Lourenço & Weitschat, 2000 (Palaeogene: 1 species)

† *Palaeoananteris* Lourenço & Weitschat, 2001 (Palaeogene: 3 species)

† *Palaeoisometrus* Lourenço & Weitschat, 2005*a* (Palaeogene: 1 species)

† *Palaeogrosphus* Lourenço, 2000*a* (Quaternary: 2 species)

† *Palaeolychas* Lourenço & Weitschat, 1996 (Palaeogene: 2 species)

† *Palaeoprotobuthus* Lourenço & Weitschat, 2000 (Palaeogene: 1 species)

† *Palaeospinobuthus* Lourenço *et al*., 2005 (Palaeogene: 1 species)

† *Palaeotityobuthus* Lourenço & Weitschat, 2000 (Palaeogene: 1 species)

*Tityus* C. L. Koch, 1836 (?Palaeogene-Recent: 6 fossil species)

† *Uintascorpio* Perry, 1995 (Palaeogene: 1 species)

Ananteridae Pocock, 1900

† *Archaeoananteroides* Lourenço, 2016 (Cretaceous: 1 species)

† *Palaeoananteris* Lourenço & Weitschat, 2001 (Palaeogene: 3 species)

† *Palaeotityobuthus* Lourenço & Weitschat, 2000 (Palaeogene: 1 species)

IURIDA Soleglad & Fet 2003 (Parvorder)

Iuroidea Thorell, 1876*b*

Iurudae Thorell, 1876*b* (Recent)

Bothriuroidea Simon, 1880

Bothriuridae Simon, 1880 (Recent)

Caraboctonoidea Pocock, 1893

Caraboctonidae Kraepelin, 1905 (Recent)

Chactoidea Pocock, 1893

† Protochactidae Lourenço, Magnani & Stockar *in* Magnani *et al*., 2022

† *Protochactas* Lourenço, Magnani & Stockar *in* Magnani *et al*., 2022 (Triassic:

1 species)

Chactidae Pocock, 1893

† *Araripescorpius* Campos, 1986 (Cretaceous: 1 species)

*Chactas* Gervais, 1844 (Quaternary–Recent: 1 fossil species)

† Palaeoeuscorpiidae Lourenço, 2003

† *Archaeoscorpiops* Lourenço, 2015*a* (Cretaceous: 1 species)

† *Burmesescorpiops* Lourenço, 2016 (Cretaceous : 1 species)

† *Palaeoeuscorpius* Lourenço, 2003 (Cretaceous : 1 species)

Euscorpiidae Laurie, 1896

† *Eoeuscorpius* Kühl & Lourenco, 2017 (Palaeogene: 1 species)

Scorpiopidae Kraepelin, 1905 (Recent)

Superstitioniidae Stahnke, 1940 (Recent)

Troglotayoscidae Lourenço, 1998 (Recent)

Belisariidae Lourenço, 1998 (Recent)

Typhochactidae Mitchell, 1971 (Recent)

Akravidae Levy, 2007 (Recent or subfossil?)

Hadruroidea Stahnke, 1974

Hadruridae Stahnke, 1974 (Recent)

Vaejovoidea Thorell, 1876*b*

Vaejovidae Thorell, 1876*b*

Scorpionoidea Latreille, 1802

Diplocentridae Karsch, 1880*b* (Recent)

Hemiscorpiidae Pocock, 1893

Lourenço (2018) and Lourenço & Velten (2021*a)* retained Protoischnuridae as a valid family

= † Protoischnuridae Carvalho & Lourenço, 2001

† *Cretaceoushormiops* Lourenço, 2018 (Cretaceous: 1 species)

† *Cretaceousopisthacanthus* Lourenço *in* Lourenço & Velten, 2021 (Cretaceous:

1 species)

† *Protoischnurus* Carvalho & Lourenço, 2001 (Cretaceous: 1 species)

Heteroscorpionidae Kraepelin, 1905 (Recent)

Hormuridae Laurie, 1896 (Recent)

Scorpionidae Latreille, 1802

† *Mioscorpio* Kjellesvig-Waering, 1986 (Neogene: 1 species)

† *Sinoscorpius* Hong, 1983 (Neogene: 1 species)

Rugodentidae Bastawade *et al*., 2005 (Recent)

Urodacidae Pocock, 1893 (Recent)

SCORPIONES *incertae sedis*

† *Brontoscorpio* Kjellesvig-Waering, 1972 (Devonian: 1 species)

† *Eramoscorpius* Waddington, Rudkin & Dunlop, 2015 (Silurian: 1 species)

† *Gondwanascorpio* Gess, 2013 (Devonian: 1 species)

† *Hubeiscorpio* Walossek, Li & Brauckmann, 1990 (Devonian: 1 species)

† *Liassoscorpionides* Bode, 1951 (Jurassic: 1 species)

† *Palaeomachus* Pocock, 1911(Carboniferous: 1 species)

† *Permomatveevia* Dammann, 2017 (Permian: 1 species)

† *Titanoscorpio* Kjellesvig-Waering, 1986 (Carboniferous: 1 species)

† *Wattisonia* Wills, 1960 (Carboniferous: 1 species)
